# Vouchers for scaling up insecticide-treated nets in Tanzania: Methods for monitoring and evaluation of a national health system intervention

**DOI:** 10.1186/1471-2458-8-205

**Published:** 2008-06-10

**Authors:** Kara Hanson, Rose Nathan, Tanya Marchant, Hadji Mponda, Caroline Jones, Jane Bruce, Godlove Stephen, Jo Mulligan, Hassan Mshinda, Joanna Armstrong Schellenberg

**Affiliations:** 1 London School of Hygiene and Tropical Medicine, Keppel Street, London WC1E 7HT, UK; 2 Ifakara Health Research and Development Centre, PO Box 78373, Mikocheni, Dar es Salaam, Tanzania

## Abstract

**Background:**

The Tanzania National Voucher Scheme (TNVS) uses the public health system and the commercial sector to deliver subsidised insecticide-treated nets (ITNs) to pregnant women. The system began operation in October 2004 and by May 2006 was operating in all districts in the country. Evaluating complex public health interventions which operate at national level requires a multidisciplinary approach, novel methods, and collaboration with implementers to support the timely translation of findings into programme changes. This paper describes this novel approach to delivering ITNs and the design of the monitoring and evaluation (M&E).

**Methods:**

A comprehensive and multidisciplinary M&E design was developed collaboratively between researchers and the National Malaria Control Programme. Five main domains of investigation were identified: (1) ITN coverage among target groups, (2) provision and use of reproductive and child health services, (3) "leakage" of vouchers, (4) the commercial ITN market, and (5) cost and cost-effectiveness of the scheme.

**Results:**

The evaluation plan combined quantitative (household and facility surveys, voucher tracking, retail census and cost analysis) and qualitative (focus groups and in-depth interviews) methods. This plan was defined in collaboration with implementing partners but undertaken independently. Findings were reported regularly to the national malaria control programme and partners, and used to modify the implementation strategy over time.

**Conclusion:**

The M&E of the TNVS is a potential model for generating information to guide national and international programmers about options for delivering priority interventions. It is independent, comprehensive, provides timely results, includes information on intermediate processes to allow implementation to be modified, measures leakage as well as coverage, and measures progress over time.

## Background

Insecticide-treated mosquito nets (ITNs) are an effective and cost-effective intervention to reduce child mortality and maternal anaemia where malaria imposes an important disease burden [1-6]. Increasing ITN coverage is therefore seen as a valuable means to progress towards the Millenium Development Goals, and has received political attention through the commitment by African governments at Abuja to increase ITN coverage among vulnerable groups to 60% [7]. However, there remains considerable debate about how best to deliver nets and target subsidies, in order to achieve an appropriate balance among the objectives of equity, efficiency and sustainability [8-14]. Many ITN projects have been implemented at a very small scale, covering individual communities, or a small number of districts. While high levels of coverage have sometimes been reached, it is not clear that the same results can be achieved when the same delivery model is expanded to national scale, due to issues of managerial capacity, accessibility, and non-constant marginal costs [15,16]. Nor is it clear whether increases in coverage are sustained over time. Only a small number of ITN delivery models operate at a national scale. These include integration of ITN distribution with measles vaccination campaigns [17-19], implemented in 18 countries from 2003 to 2007 (Mark Grabowsky personal communication) and social marketing through public sector facilities in Malawi and Kenya [20,21].

**Figure 1:**
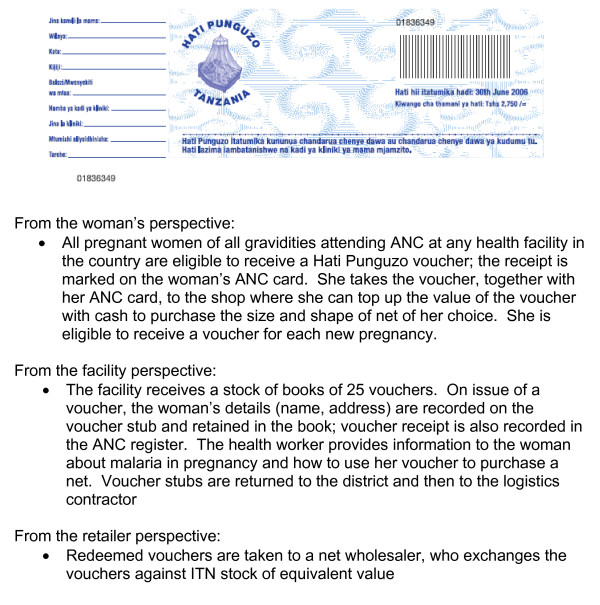
The *Hati Punguzo *scheme.

The Government of Tanzania was awarded a Round 1 grant from the Global Fund to fight AIDS, TB and Malaria (GFATM) of $19 million to scale up ITN delivery using an innovative voucher scheme, known as *Hati Punguzo *("discount card" in kiSwahili) (see Figure 1). The scheme builds on the success of an earlier discount voucher scheme which operated in the context of a social marketing programme [4,22,23]. The theoretical advantages of a voucher scheme over other modes of targeting a subsidy include 1) it reduces to some extent the burden placed on the health system to stock and distribute bulky ITNs, and to handle cash; 2) it allows women to choose their preferred size, shape and colour of net; 3) it reinforces the commercial distribution system by using it to deliver subsidised nets, which should help to improve the sustainability of programme benefits. These are important considerations in Tanzania which already has a relatively well-developed commercial distribution system for ITNs, supported through a strategic social marketing programme (SMARTNET) [22]. However, the cost and cost-effectiveness of vouchers in achieving and sustaining high levels of coverage in vulnerable groups has yet to be demonstrated.

Under the Tanzania National Voucher Scheme (TNVS) every pregnant woman is eligible to receive a voucher at her first antenatal care visit. *Hati Punguzo *is available in all reproductive and child health facilities that operate the national government recordkeeping system for registering new clients, "*MTUHA"*. This includes some private facilities. Specialist referral clinics in hospitals are excluded. The value of the voucher was initially fixed at TSh 2750 (approximately US$2.45), and was increased in 2006 to TSh3250. The woman can use the voucher as part-payment for an ITN (a net packaged with a single-treatment sachet of insecticide) from any participating retailer. In 2005/6, the retail price of an ITN was TSh3000-4000, implying a subsidy of 70–90%, and a top-up payment of TSh 250–1000 (US$0.21–0.87). Monthly household income (including the value of own-production) in rural areas was estimated to be $90 in 2000/1[23]. A second voucher was introduced in 2007, given to all infants at the time of measles vaccination, funded by the US President's Malaria Initiative (PMI).

**Figure 2:**
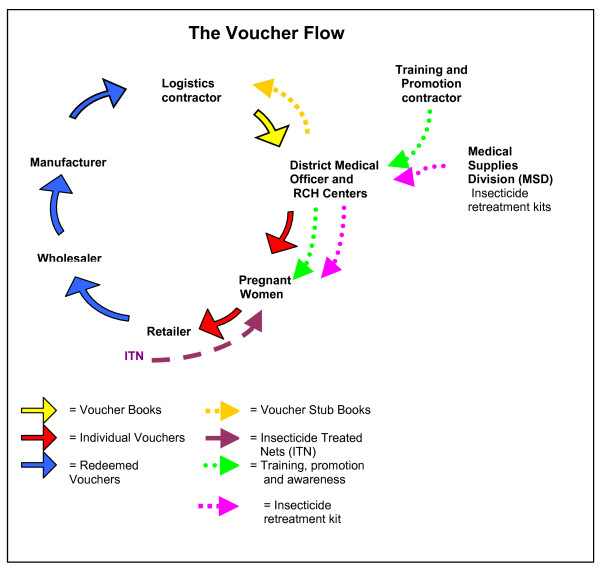
The voucher flow.

Implementation of the scheme is through a partnership of the National Malaria Control Programme, the district health offices, and 3 non-governmental organisations (NGOs) contracted to the Ministry of Health: one for logistics (Mennonite Economic Development Associates) and two for training and promotion (Care and World Vision). Responsibility for voucher supply, distribution and redemption lies with the logistics contractor, which procures vouchers with a number of security measures aimed to reduce the likelihood of counterfeit. Vouchers are delivered by the logistics contractor to district level, and through the District Medical Officer (DMO) to Reproductive and Child Health (RCH) facilities. RCH facilities distribute them to pregnant women who then redeem the vouchers for ITNs at local appointed retailers. Redeemed vouchers are returned to wholesalers and then to manufacturers in exchange for new stock. Cash is provided against vouchers only at the very top of the system, by any of the four manufacturers or a limited number of large wholesalers. This is to minimise the misuse of vouchers. The flow of vouchers and ITN products is illustrated in Figure 2. A parallel system is used to supply insecticide re-treatment kits free of charge to children attending vaccination clinics at 3 months and 9 months, to encourage regular retreatment of nets. The Medical Stores Department (an autonomous agency of the Ministry of Health and Social Welfare) supplies insecticide retreatment kits directly to the districts through the normal distribution channels used for drugs and other medical supplies.

A phased roll-out of the programme was defined by the implementing partners. The first voucher was distributed in October 2004, and by May 2006 all districts in the country had been reached and were delivering vouchers routinely through ANC services.

Because there is little evidence on the impact of ITN delivery systems operating at scale, a comprehensive approach to monitoring and evaluation was adopted. Implementing partners needed timely information on effects and implementation processes, and the international public health community needs information about the effectiveness and cost-effectiveness of using vouchers to deliver ITNs. The Ifakara Health Research and Development Centre (IHRDC) and the London School of Hygiene and Tropical Medicine (LSHTM) were contracted by the Ministry of Health and Social Welfare to conduct independent monitoring and evaluation. The objectives of this paper are to describe how the approach to M&E was defined; to present the underlying conceptual framework; to describe in detail the methods that were used, and to highlight ways in which the M&E was able to respond to emerging issues. The results of the evaluation, including both changes in ITN coverage and intermediate process indicators, will be published separately.

## Methods

The M&E methods were developed at a meeting of TNVS partners in November 2003, and refined in a second meeting a year later in November 2004. Both meetings included stakeholders from the National Malaria Control Programme, groups involved in implementing ITN activities in Tanzania, and researchers. Because of the novelty and complexity of the intervention, involving multiple partners and depending on both the health system and the retail sector, a comprehensive and multidisciplinary approach to monitoring and evaluation was agreed. By "evaluation", we mean the measurement of programme effects (see below for a description of these), while we use the term "monitoring" to describe the measurement and assessment of intermediate processes. Measuring health impact (in terms of mortality and morbidity prevented) was beyond the scope of the resources available; the health impact of ITN use on morbidity, mortality and anaemia had been demonstrated under effectiveness conditions in an earlier study in Tanzania[2,4,6]. Furthermore, the evaluation indicators agreed with GFATM were also based on intermediate outcomes, with health impact imputed from published estimates of intervention effectiveness.

Programme effects were investigated over five main domains: (1) ITN coverage among target groups, (2) provision and use of RCH services, (3) "leakage" of vouchers, (4) the commercial ITN market, and (5) cost and cost-effectiveness of the scheme. The principle of triangulation was adopted, in which data would be collected from multiple sources of information, compared, and reasons for divergences explored. Table 1 shows the evaluation domains, the core indicators, and the data sources for each. Coverage of "any net" and "effectively treated net" were distinguished. ITN coverage in pregnancy was calculated over currently pregnant women, while the denominator for voucher coverage was completed pregnancies. English versions of all of the questionnaires used are available as additional files 1 to 8.

**Table 1:** Evaluation domains and data collection methods: triangulation of data sources

Evaluation domain	Indicator(s)	Household survey	Facility survey	Exit survey	Focus group discussions and in-depth interviews	Retail census	Voucher tracking	Cost analysis
Coverage of target groups (ownership, use)	Household ownership of at least one net/ITN; Individual slept under a net/ITN the night prior to the survey*	X	X	X				
Provision and use of RCH services, including voucher scheme	Currently pregnant woman/recently pregnant woman**attended ANC; Mean weeks of gestation at time of first ANC visit; Received a voucher; Received 1 dose of SP as IPTp; Received 2 doses of SP as IPTp	X	X	X	X		X	
Leakage of vouchers	% of voucher recipients who could be identified, interviewed, and confirmed they received a voucher				X		X	
Impact on ITN market	Percent of wards with at least one retail source of ITNs, insecticide	X				X		
Cost and cost-effectiveness	Cost per voucher delivered Cost per ITN delivered	X						X

* ITN use measured in all household members, currently pregnant women, children under 5 years.

** Indicators of voucher coverage were calculated for both currently pregnant women and for women who had a live birth in the 12 months preceding the survey.

ITN = Insecticide-treated net; RCH = Reproductive and child health; ANC = Antenatal care; SP = sulphadoxine-pyrimethamine; IPTp = Intermittent preventive treatment in pregnancy

**Figure 3:**
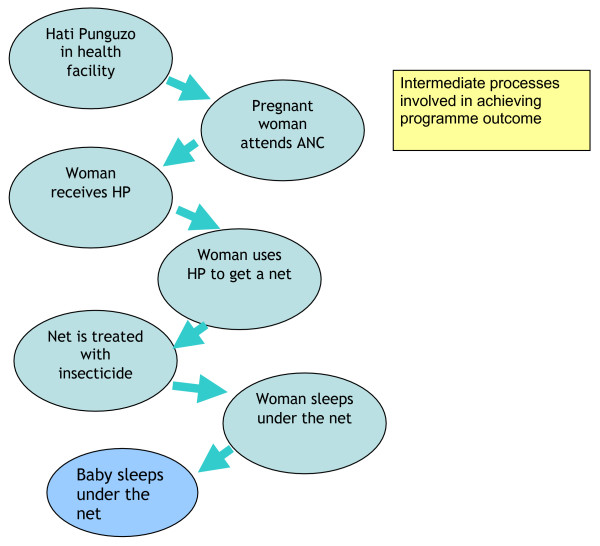
Intermediate processes involved in achieving programme outcome.

A key principle of the M&E approach was that it should also provide information which would shed light on implementation issues, in order to inform programming choices, and therefore intermediate processes as well as outcomes (measured as ITN coverage) should be investigated. Figure 3 shows the intermediate steps required to achieve the desired outcome of ITN use by pregnant women and infants. Indicators therefore included intermediate processes, such as the proportion of facilities stocking vouchers, the proportion of women receiving a voucher at their ANC visit, and voucher redemption rates. Regular interaction with implementers was anticipated, with feedback at each point when new results from each of the M&E approaches were available.

## Results

Because the "operational unit" of the programme is the district, M&E activities were focused at this level. Within the budget available it was possible to concentrate the M&E activities in a nationally representative sample of 21 focal districts (about 1 in 6 of all districts in mainland Tanzania). These were randomly selected from a list of 113 from the President's Office for Regional and Local Government, stratified by *Hati Punguzo *launch date. Information about launch date was obtained from the Training and Promotion Contractor in January 2005.

### Household surveys

A "baseline" household survey took place in June-August 2005 and follow-up surveys in June-August 2006 and 2007. The primary purpose was to measure the effect of the voucher scheme on ITN use among pregnant women and children under five years, together with providing information on intermediate processes (voucher receipt and use) and use of RCH services including the voucher scheme.

Sample size was set to estimate ITN use in each district among children under 5 and children under 1 with reasonable precision (± 10% for under-fives and ± 20% for under 1 s). In each district 10 clusters of 30 households were selected (n = 6300 households in total). A cluster was sampled in two stages: first 10 wards within each district were selected with probability proportional to ward population. Within each chosen ward, one sub-village (*kitongoji*) was selected using simple random sampling. This constituted the primary sampling unit. Within each selected kitongoji, 30 households were chosen using a modified EPI-type sampling procedure which ensured an equal chance of selection for each household. In subsequent rounds, PSUs were selected randomly from within the same wards. An interviewer-administered questionnaire was used to collect information from the household head (number of residents, household characteristics, ITN coverage of all household members), all women aged 15–49 (health service use for current and recent pregnancies, ITN use in pregnancy), and carers of all children aged 0–59 months (ITN use).

The surveys took place during July-August, after the rainy season and immediately after the peak malaria transmission period. The infrastructure of the country poses considerable logistical challenges for mounting such large surveys during the rains.

### Facility surveys

Two rounds of facility surveys, accompanying the household surveys, were undertaken to provide information about the effect of the scheme on RCH service provision. The facility questionnaire was administered in the health facility (public or private) which served each cluster of households, giving a total of 10 facilities per district (n = 210 facilities in total). Information was collected on equipment, supplies and provision of antenatal services, including voucher distribution, and on the conduct and content of health education and health promotion activities.

### Health facility user survey

Structured interviews with health facility users were undertaken at the time of both rounds of the facility survey, to provide information about pregnancy history, use of RCH services during pregnancy, voucher knowledge and use, ITN use and knowledge of malaria in pregnancy. A short questionnaire was administered to the first 7 women to leave the facility on the day of the survey.

### Group discussions and in-depth interviews

Community and provider perspectives on the scheme were explored through group discussions and in-depth interviews. These took place in two rounds, with each following the analysis and preliminary interpretation of the household and facility survey data.

In each of the two rounds (Jan-Feb 2006 and Oct 2006) data were collected in purposively selected districts from among the 21 M&E districts in which the scheme had been operating for at least 12 months. In each district, two health facilities (one rural and one urban) were selected. In each facility, group discussions were undertaken with health workers involved in the delivery of RCH services and interviews with pregnant women waiting to attend a RCH clinic. Group discussions were conducted among women, and separately among men, who lived in close proximity to the selected health facilities as well as in communities located towards the periphery of the catchment area of each health facility. In-depth interviews with the district malaria focal person and District Medical Officer (DMO) were undertaken in each district. The second round did not include group discussions with users.

### Retail audit

Data on ITN availability and retail prices were collected from two rounds of a survey of a random selection of shops. In each district, 30% of wards were selected, stratified by whether they contained a "major" or "non-major" trading centres. All shops in the selected wards were visited and a structured questionnaire administered containing questions about availability of net products, net prices and participation in the voucher scheme.

### Voucher tracking

Voucher tracking [24] was used to estimate the degree of "leakage" from the voucher scheme and to provide additional information about voucher redemption and net use. For the purposes of this study, leakage refers to inappropriate use of the voucher and is defined in terms of non-target groups receiving vouchers, and vouchers being used to purchase items other than ITNs. Two rounds of voucher tracking were undertaken in the M&E districts.

In each round, a random sample of redeemed vouchers from among the 21 M&E districts was selected. In the first round, the sampling frame was restricted to districts which had launched by February 2005, so that enough time would have elapsed for the vouchers to be issued from the health facility, redeemed by the woman, and worked their way back up the distribution system to the logistics contractor (see Figure 1). At the time of net purchase, both the voucher and the woman's ANC card are required. Information recorded on the voucher by the shopkeeper (ANC card number, woman's name, shop registration number, shopkeeper name, region, district, division, ward, date, size and type of net purchased) and information from the voucher stub was extracted and used, together with any additional information that could be collected from records at the issuing facility, to locate the recipient. If the woman was located, a short questionnaire was administered with questions about voucher receipt and use. Those who could be identified but not interviewed (for example because they had travelled) were also recorded, together with those who could not be located.

### Cost study

Economic and financial costs of the voucher scheme were estimated using a standard methodology developed for costing ITN distribution systems [25].

### Data processing and analysis of household, facility and user survey data

Data for all three questionnaires were entered into handheld computers at the point of data collection[26]. Cleaned data were analysed according to an analytical plan that detailed indicators and agreed definitions. Stata v 9.0 software [27] software was used for analysis to obtain estimates for the indicators correctly controlling for the survey design, overall and at the district level where required.

In the facility user, household and voucher tracking surveys socioeconomic status was measured as an index made up of education of household head, housing conditions, asset ownership, and whether the house was rented or not [28]. Weights for the variables were derived using principal components analysis, and the index was generated from the first principal component, which summarises the largest amount of information common to the variables. In all cases, households were divided into 5 equal sized groups (quintiles) according to their value of the continuous socioeconomic status score generated by the principal components analysis.

Triangulation of results was undertaken through systematic comparison and presentation of indicators from multiple sources. Where indicators were measured over different populations, this included an assessment of whether differences were consistent with the different nature of the populations.

### Experience of implementing the M&E plan

**Figure 4:**
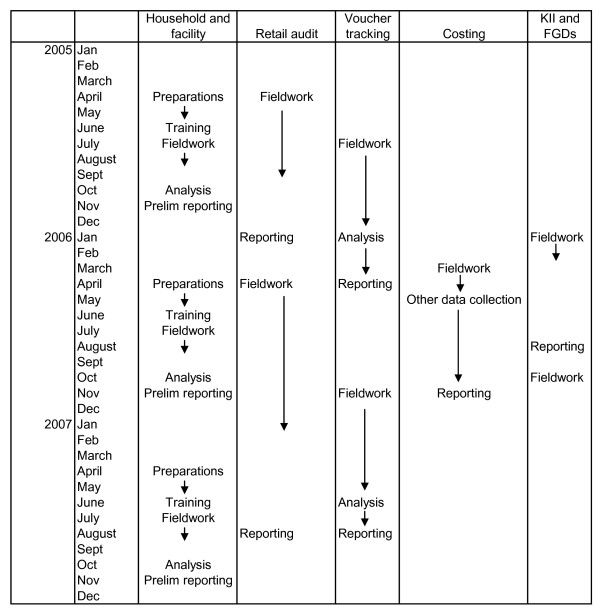
Schedule of fieldwork and reporting (actual).

From the outset, the M&E was implemented on a national scale. Data collection, analysis and feedback operated as a continuous process. Preparations for the first household and facility survey began around February 2005, piloting and pretesting took place in May/June 2005, data collection during July-August, and preliminary results from the first survey round were presented to implementation partners in early November 2005. A similar timeframe applied to the 2006 and 2007 household and facility surveys. The first round of retail audit data collection commenced in April 2005 and took six months. Preliminary results were presented to TNVS partners in early 2006. The first round of voucher tracking started in late July 2005, and the first results were presented in March 2006. The cost analysis was conducted during 2006. In effect, the evaluation team met with implementers at intervals of between 3–4 months in the first 2 years. Figure 4 shows the schedule of fieldwork and reporting for each of the main data collection methods.

The regular exchange over findings appears to have been valued by implementers, and has been used to modify implementation strategies (Table 2). The perceived value of the evaluation activities can be seen in the inclusion of a significant evaluation component and budget in subsequent funding proposals submitted by the National Malaria Control Programme (NMCP) to both PMI and GFATM. In addition, successive quantitative and qualitative data collection rounds have included additional questions requested by the programme managers (Table 2). A particular challenge posed by the implementer interest in evaluation data has been around managing the desire for an ever increasing level of district level detail. For instance, the sample size for the household survey is powered to estimate with reasonable precision ITN coverage among children under 5 years at the district level. There was a debate before the 2006 and 2007 surveys about the feasibility and value of revisiting the sample size to allow district-level estimates of ITN coverage in pregnant women, and the socioeconomic distribution of coverage in this target group. In the end, the original sample size was retained.

**Table 2:** Implementation issues identified from 2005 surveys

Issue identified	Evaluation Response	Implementer Response
Relatively low levels of voucher coverage	Qualitative investigation with facility staff to identify reasons for not distributing vouchers	Redevelop training messages for facility workers
Stock-outs of vouchers and antenatal cards required for issuing of voucher	In-depth facility level analysis	Work with the Medical Supplies Division and MEDA to improve supply chains
Relatively low levels of voucher coverage in most geographically isolated clusters	Survey instruments modified in subsequent rounds to include questions about use of outreach services for ANC, and the interventions received.	Develop mechanisms for outreach providers to distribute vouchers
Relatively low levels of retreatment of bednets	Qualitative investigation to identify what voucher recipients understand about retreatment kits	Amend insecticide treatment messages to respond to user knowledge and perceptions
Low knowledge of voucher value	Qualitative investigation into understanding of the value of the value	Develop IEC materials to address voucher value and top-up

The total cost of the M&E for the first two years was approximately $1 million, of which $805,000 was allocated from the Global Fund grant. In total, 4% of the GFATM round 1 grant of $19 m was allocated to monitoring and evaluation. The largest single activity was the household and facility survey conducted in each year, estimated at $315,000 per round (approximately $15,000 per district per round).

## Discussion

The dearth of evidence about the impact of development interventions, including those addressing public health issues, is increasingly recognised [29] and is all the more important given opportunities for change provided through the large sums of money now being channelled through global health initiatives such as the Global Fund.

Bryce and Victora[30], writing in the context of the multi-country evaluation of Integrated Management of Childhood Illness, identify a number of methodological issues arising in large-scale programme evaluations, many of which have been relevant to the TNVS evaluation. These include issues of methodology (the definition of scope of the evaluation, the selection of study districts, the protection of objectivity in interpretation); the way in which feedback has been provided to implementers and policymakers; and the provision of adequate resources to support both the analysis and interpretation of findings and their communication.

In light of these lessons, six features of the TNVS M&E deserve comment. The first is its independence. While programme managers and implementers were involved in the design of the overall M&E strategy, the execution, analysis, interpretation and reporting of the M&E results were entirely independent.

The second feature is its breadth. Because TNVS is implemented through both the health system and the retail sector the evaluation domains were defined to include outcomes that are broader than ITN coverage alone (e.g. ANC attendance, receipt of intermittent preventive treatment (IPT)) and outcomes accruing to a broader range of actors (health facilities and shops); the cost analysis was conducted from the societal perspective to capture costs incurred by voucher users as well as providers.

A third feature is timely reporting of results to programme managers. Regular feedback of findings has helped implementers identify problems early and to revisit their strategies where necessary (Nick Brown, personal communication). This has also created a demand for M&E information from programme managers and they have continued to advocate for the inclusion of comprehensive M&E in subsequent funding applications to the Global Fund and PMI.

A related feature has been the inclusion of intermediate processes. Such information is important in supporting inference about programme impact on ITN coverage in a non-random design, but additionally can be used by programme implementers to adjust their strategies because it provides richer information about the "hows and whys" of programme effects.

A fifth distinguishing feature of this M&E plan is the attempt to actively measure leakage or misuse of vouchers through voucher tracking. Misuse is a problem for many different public sector resources but is difficult to measure and data are scarce [31]. Interpreting measured levels of leakage from a voucher scheme is particularly difficult since there is no clear threshold of "acceptable" levels of leakage. It is important to set leakage levels in the context of leakage of other commodities through other delivery channels, yet few comparator estimates exist.

The ability to monitor changes in outcome measures over time is the final distinguishing feature of the TNVS M&E. First, many programmes measure outcomes soon after the intervention and are unable to determine whether the outcomes have been sustained. For instance, monitoring of the effects of mass integrated vaccination and ITN distribution campaigns took place 6 months after implementation in Zambia [17]; 5 months in Ghana [18], 1 month in Togo [19], and 3 months in Tanzania [32]. The approach here of annual household and facility surveys allows some assessment of the degree to which coverage increases are maintained over time. The second element is the effect of time in allowing a delivery system to "bed down" and become institutionalised within the health system. This is particularly important when an intervention is integrated with routine service delivery such as antenatal care. The stratification of districts by launch date, together with multiple rounds of data collection, will allow some investigation of the degree to which these systems are effectively institutionalised.

The TNVS M&E stopped short of measuring health impact and focused on measuring process indicators and outputs. We felt this to be justified given the resources available and the existing evidence about efficacy and effectiveness of ITNs. A strength of the design has been the ability to examine certain outputs and processes at the district level, allowing some comparison of achievement across districts (though in some cases limits are imposed by the sample size in each district). However, it has been challenging to directly compare the results with other programmes, because of differences between countries in the timing and scale of ITN delivery interventions, and also importantly, in evaluation domains, methods, and timeframes. Since comparisons of effectiveness and cost-effectiveness are unavoidably relative (how did one approach do compared with another), the lack of strictly comparable evaluation data from another national scale programme makes it difficult to reach firm judgements about whether this particular ITN distribution system was able to achieve equity, efficiency, sustainability, and appropriate levels of opportunity cost in terms of demands on managerial capacity and health systems, etc.

A further question is whether it is feasible to continue to research ITN coverage and its determinants on such a large scale. One option would be to use existing national surveys, such as the DHS, to provide information about progress. The Tanzanian programme managers have continued to advocate for large scale and regular M&E. We believe this is because of the information provided on implementation processes, and also because it provides a degree of validation of the routine information, such as estimates of the voucher redemption rate, which is collected by programme implementers through their project information systems.

## Conclusion

Resources to scale up ITN delivery systems have increased dramatically through the new global initiatives, particularly the Global Fund and the PMI. However, governments applying for these funds have very limited evidence on which to draw in selecting among different delivery strategies or combinations of strategies. Bennett and others [33] have called for a "scaling up of HIV/AIDS evaluation". Similar efforts are needed to expand the evidence base for delivery of ITNs and other critical malaria control interventions, such as artemesinin-based combination therapy (ACT), where innovative delivery mechanisms are essential to attain high levels of coverage. The Government of Tanzania has been supportive of the need to evaluate carefully and comprehensively their efforts to scale up ITNs. They have allocated a share of their Global Fund grant to monitoring and evaluation. The evaluation is conducted independently through a partnership of local and international research organisations. And regular close interaction takes place between the implementers and the evaluators, to ensure that the lessons of evaluation are translated promptly into modifications and improvements to the programme. This model deserves further attention from both the Global Fund and from recipient countries.

## Availability

Documents relating to the Tanzania Round 1 proposal can be found at:  (accessed 20 May 2008)

## Competing interests

The authors declare that they have no competing interests.

## Authors' contributions

KH and RN are co-principal investigators. KH, RN, TM, CJ, HM and JAS designed the study. Design of data collection instruments was undertaken by RN, TM, HM, CJ, JB, GS, and JM. KH drafted the paper. All authors critically revised the paper and approved the final manuscript. KH is the guarantor of the paper.

## Pre-publication history

The pre-publication history for this paper can be accessed here:



## Supplementary Material

Additional file 1 Household questionnaire. Click here for file

Additional file 2 Facility questionnaire. Click here for file

Additional file 3 Facility users survey questionnaire. Click here for file

Additional file 4 Community group interview topic guide. Click here for file

Additional file 5 Health worker interview guide. Click here for file

Additional file 6 Retail survey questionnaire. Click here for file

Additional file 7 Voucher tracking survey, Form 1 (recipient found). Click here for file

Additional file 8 Voucher tracking survey, Form 2 (recipient not found). Click here for file
