# Supplementary material for: Vouchers for scaling up insecticide-treated nets in Tanzania: Methods for monitoring and evaluation of a national health system intervention
Source: BMC Public Health. 2008 Jun 10;8:205. doi: 10.1186/1471-2458-8-205 (PMC2442068; doi:10.1186/1471-2458-8-205)
Supplement: Additional file 1 — Household questionnaire. [file 1471-2458-8-205-S1.pdf]

Tanzania National Voucher Scheme for insecticide treated nets
HOUSEHOLD SURVEY

Ifakara Health Research and Development Centre *in collaboration with*
Ministry of Health, Tanzania and London School of Hygiene and Tropical Medicine

Module 1. Household questionnaire

H1	District:<br>(drop down)	|__| district
H3	Ward (drop down)	|__|__|__|__|__|__|__|__|__| ward
H5	Sub village (drop down)	|__|__|__|__|__|__|__|__|__|__|__|__|
H6	Cluster no	|__|__| cluster
H7	Household no	|__|__| hhno
H8	Interviewer initials	|__|__| int
H9	Date (dd/mm/yyyy)	|__|__|/|__|__|/|__|__|__|__| date
H10	Name of household head	|__|__|__|__|__|__|__|__|__|__|__|__| hhname

H11	**Have you read him/her the consent form?**<br>1= yes<br>2=no	|__| readconsent
H12	Does the respondent agree?<br>1=Yes<br>2=No **IF NO END INTERVIEW HERE**	|__| respagree
H13	Who is the respondent?<br>*1=Household head*<br>*2=Representative*	|__| whoresp

Tanzania National Voucher Scheme for insecticide treated nets
HOUSEHOLD SURVEY

Ifakara Health Research and Development Centre *in collaboration with*
Ministry of Health, Tanzania and London School of Hygiene and Tropical Medicine

H14. Please can I ask the names of all the people in your household? **Start with the head of household and older people**

Number of the person	*Name of the person*	*Sex* 1=M 2=F	*Date of birth* (dd/mm/yyyy)	*Woman between 15 and 49 years?* 1 = Yes 2 = No **(born between 1956 and 1991)**	*Child under 5 years?* 1 = Yes 2 = No **(Born 2001 to present)**	*Child under 2 years?* 1=Yes 2=No **(Born 2005 or 2006)**	*Name of the mother/guardian* ***(under five years only)***	*Who cares for this child? 1=Mother 2=Guardian*	*What does he/she do?* ***(see codes below) (Answer if aged over 14 years i.e. born in 1990 or earlier) Probe for any second occupation***	*Completed years of education*	*Did the person sleep in the household last night?* 1 = Yes 2 = No	***Is this the person who is being interviewed for the household questionnaire?*** **1=yes 2=no**	***Is there anybody else in the household?***

Occupation codes:

1=Farming, fishing, forestry
2=Mining
3=Non-agricultural day-labour
4=Professional/clerical
5=Service (e.g. repair, hairdressing, tailoring)
6=Selling (E.g. vegetables, products
7=Business
8=Domestic worker
9=Student
10=Not employed

Tanzania National Voucher Scheme for insecticide treated nets
HOUSEHOLD SURVEY

Ifakara Health Research and Development Centre *in collaboration with*
Ministry of Health, Tanzania and London School of Hygiene and Tropical Medicine

About the household

H15	Do you rent this house?<br>*1 = yes*<br>*2=no*<br>*3=Other (specify)*	|__| rent<br>otrent
H16	What kind of toilet facilities does your household have?<br>*1 = Flush toilet*<br>*2 = Pit toilet/latrine*<br>*3 = No facility/bush/field*	|__| toilet<br>ottoilet
	In this household is there anyone who owns:	
H17	Radio<br>*1= yes*<br>*2= no*	|__| radio
H18	Bicycle<br>*1= yes*<br>*2= no*	|__| bike
H19	Mobile phone<br>*1=yes*<br>*2=no*	|__|

H20	In this house are there ducks or chickens? How many? *(write the number; 999 if respondent does not know)*	|__|__|__|
H21	Do you have animals in this household like goat, sheep or cattle-how many? *(write the number; 999= she / he do not know.*	|__|__|__|
H22	Is the house connected to electricity<br>*1= yes*<br>*2= no*	|__| electric
H23	What is the main material of the roof:<br>*1= Iron sheets or tiles*<br>*2= Thatch/grass or leaves*<br>*3= Other (explain)*	|__| roof<br>otroof

H25	Have you heard of Hati Punguzo, the discount voucher programme to buy a mosquito net at a cheaper price?<br>*1=Yes*<br>*2=No* ***(SKIP TO H28)***	|__| heardvouch
H26	If yes, where did you first hear about the discount voucher?<br>*1 = RCH or health facility*<br>*2 = Shop*<br>*3 = Family member*<br>*4 = Neighbour*<br>*5 = Radio*<br>*6= Performance by theatre group or roadshow*<br>*7 = Others*<br>*8 = Village government*<br>*8a = Newpaper*	|__| heardwhere<br>otheardwhere

	9= I don't know	
H27	Who is eligible to receive a voucher?<br>*1= Pregnant women*<br>*2= Child < 1*<br>*3= Pregnant woman and child*<br>*4=Other (specify)*	|__| eligible<br>oteligible<br>________________<br>________________<br>________________
H27a	Can you tell me the value of HP? *(write the amount or write 0 if doesn't know)*	|__|__|__|__|
H47	Do you ever listen to the radio?<br>*(1)Yes (2)No*<br>***If NO go to H54***	|__| hhradever
H48	How frequently do you listen to the radio?<br>*1) Almost every day,*<br>*2) At least once a week,*<br>*3) less than once a week?*<br>***If (b) or (c) go to H50***	|__| hhradfreq
H49	How many times a day do you listen to the radio?<br>***(Write number)***	|__| hhradday
H50	At what times do you listen to the radio?<br>*1)morning 2)afternoon 3)evening 4) night*	|__| hhradtime
H51	Which radio stations do you listen mostly?<br>*1) Radio free Afrika*<br>*2) Radio Tanzania (Radio ya Taifa*<br>*3) Radio Uhuru*<br>*4) Radio Clouds*<br>*5) Radio Aboud*<br>*6) Radio Tumaini*<br>*7)Radio Ukweli*<br>*8) PRT*<br>*9) Others (specify)*	|__| hhradwhich
H52	*If H51==9 (others) specify*	________________<br>hhradoth
H53	Which radio programs you most like to listen?<br>*1) news bulletin*<br>*2) sports*<br>*3) films*<br>*4) Music*<br>*5) Others)*	|__| hhradprog
H54	Do you ever watch community drama or road shows performances?<br>*(1)Yes (2)No*<br>***If (2)No, go to H57***	|__| hhdraever
H55	Last year, how many times did you watch these shows?	|__|__| hhdranum

	(Write number)	
H56	Did you get any HATI PUNGUZO message from these performances?<br>*1)Yes 2)No*	|__| hhdrahp
H57	Do you usually read newspapers?<br>*1)Yes 2)No*<br>***If 2)No, go to H59***	|__| hhnews
H58	How frequently do you read newspapers?<br>*1) Almost every day,*<br>*2) At least once a week,*<br>*3) Less than once a week?*	|__| hhnewsfreq
H59	Do you usually read posters at health facilities?<br>*1)Yes 2)No*<br>***If 2) No, go to H61***	|__| hhpostusu
H60	How frequently do you read these posters?<br>*1) Almost every time when seen*<br>*2) Only once and not repeated once seen*<br>*3) Don't remember*	|__| hhpostfreq
H61	In the past month, have you heard or seen any messages about HATI PUNGUZO?<br>*1)Yes 2)No*<br>***If 2)No, go to H28***	|__| hhmthhp
H61	*Where did you hear or see these messages?*<br>***(do not ask but wait for respondent to mention)** (1=Yes, 2=No)*<br>a) RADIO....................... ……………………………….1 2 hhmthrad<br>b) NEWSPAPERS........... ……………………………….1 2 hmthnews<br>c) MAGAZINES .... ……………………………….1 2 hhmthmag<br>d) BILLBOARDS .... ……………………………….1 2 hhmthbrd<br>e) FOOTBALL MATCH..... ……………………………….1 2 hhmthfoot<br>f) CONCERT................. ……………………………….1 2 hhmthcon<br>g) COMMUNITY RALLY / . ……………………………….1 2 hhmthral<br>h) ROAD SHOW .... ……………………………….1 2 hhmthroad<br>i) TEE SHIRTS/CAPS ..... ……………………………….1 2 hhmthroad<br>j) MOBILE VIDEO .... ……………………………….1 2 hhmthvid<br>k) TIRE PROTECTOR...... ……………………………….1 2 hhmthtire<br>l) CALENDAR................ ……………………………….1 2 hhmthcal<br>m) POSTER .... ……………………………….1 2 hhmthpost<br>n) STICKER................... ……………………………….1 2 hhmthstick<br>o) CLINIC .... ……………………………….1 2 hhmthclin<br>p) OTHER………………………………………..…. ... 1 2 hhmthoth	
H28	How many mosquito nets does your household have?<br>***If "0" SKIP TO M1***	|__|__| numnets

		Net # 1	Net # 2	Net # 3
H29	**Is the net observed?**<br>*1 =Yes*<br>*2 = No*	|__| seennet1	|__| seennet2	|__| seennet3
H30	How long ago did your household obtain the	|__|__| whenbought1	|__|__| whenbought2	|__|__| whenbought3

	mosquito net?<br>*(Months ago)*<br>***If more than 3 years, indicate 96***<br>***Don't know write 99***			
H31	Was the net purchased using a voucher/Hati Punguzo?<br>*1=Yes*<br>*2=No*<br>*9=Don't know*	|__| usedvouch1	|__| usedvouch2	|__| usedvouch3
H31a	Did you receive the net (free during the free nets distribution to children)<br>**FOR TANDAHIMBA AND NACHINGWEA ONLY**<br>*1=Yes 2=No*			
H32	From what kind of source did your household obtain the mosquito net?<br>*1=Shop*<br>*2= Hawker (mobile)*<br>*3= Health facility*<br>*4= Government or NGO project*<br>*5= Other*<br>*6= Shifting market (gulio)*<br>*7= Gift*<br>*9= Don't know*	|__| wherebought1<br>otwherebought1	|__| wherebought2<br>otwherebought2	|__| wherebought3<br>otwherebought3
H33	What size is the net?<br>*1=3.5X6*<br>*2=4X6*<br>*3=6X6*<br>*4=Other*	|__| size1<br>otsize1	|__| size2<br>otsize2	|__| size3<br>otsize3
H34	What price was paid for this net?<br>***(write "0" if gift)***<br>***(write "9999" if don't know)***	TSh. __________<br>Paidnet1	TSh. __________<br>Paidnet2	TSh. __________<br>Paidnet3
H36	Did the net come packaged with a sachet of insecticide?<br>*1=Yes*<br>*2=No*<br>*9=Don't know*	|__| withngao1	Withngao2	|__| withngao3
H37	Has this net ever been treated?<br>*1=Yes*<br>*2=No* ***(SKIP TO H39)***<br>*3=Not sure* ***(SKIP TO H39)***	|__| evertreat1	|__| evertreat2	|__| evertreat3
H38	When was the last time the net was treated?<br>*(month/year)*<br>**(If don't know, write 1 July 2009)**	|__|__|__|__|	|__|__|__|__|	|__|__| |__|__|__|__| lasttreat3

		lasttreat1	lasttreat2	
H39	Did anyone sleep under the mosquito net last night?<br>*1 = Yes*<br>*2 = No* ***(SKIP TO H41)***<br>*3 = Not sure* ***(SKIP TO H41)***	|__| sleeplstnt1	|__| sleeplstnt2	|__| sleeplstnt3
H40	Who slept under this mosquito net last night ***(record the line number of the individual(s) from the household roster H14)***	Jina__________<br>Jina ________<br>Jina ________	Name ________<br>Name ________<br>Name ________	Name ________<br>Name ________<br>Name ________
	Observe and record the condition of the net. Record the number of holes. Do not include repaired holes. If more than 10 holes of a given size, write "96"	|__|__| n1head	|__|__| n2head	_|__| n3head
H41	*Number of holes head size*			
H42	*Hand size*	|__|__| n1hand	|__|__| n2hand	|__|__| n3hand
H43	*Finger size*	|__|__| n1finger	|__|__| n2finger	|__|__| n3finger
H44	*Net too torn to count holes*<br>*1=Yes*<br>*2=No*	|__| n1torn	|__| n2torn	|__| n3torn

H46	Are these the people who did not sleep under a net last night? **(record the line numbers)**<br>***If everybody in the household slept under a net leave blank***	|__|__| |__|__| |__|__| |__|__| |__|__| |__|__| |__|__| |__|__| nosleep1<br>nosleep2<br>nosleep3<br>nosleep4<br>nosleep5<br>nosleep6<br>nosleep7<br>nosleep8

Tanzania National Voucher Scheme for insecticide treated nets
HOUSEHOLD SURVEY

Ifakara Health Research and Development Centre *in collaboration with*
Ministry of Health, Tanzania and London School of Hygiene and Tropical Medicine

Module 2: Women aged 15-49 years

M1	Name of the mother	mothername
M2	ID of the mother<br>*(District/cluster/household/person)*	|__|/|__|__|/|__|__|/|__|__| motherid

M3	Is it possible to interview the woman?<br>*1 = yes* ***(SKIP TO M5)***<br>*2 = No*	|__| intwom1
M4	Why is it not possible to interview?<br>*1=Travelled away*<br>*2 = Sick*<br>*3 = Other*	|__| whynot1<br>otwhynot1

M5	Have you read her the consent form?<br>*1= yes*<br>*2=no*	|__| readconsent
M6	Does the mother agree?<br>*1=yes*<br>*2=no* ***IF NO, END INTERVIEW HERE***	|__| motheragree

Interviewer: If the eligible woman was the respondent to Module 1, skip to M10

M7	Have you heard about the discount voucher programme for buying a net at a cheaper price?<br>*1=Yes*<br>*2=No* ***(SKIP TO M10)***	|__| heardvouch
M8	If yes, where did you first hear about the discount voucher?<br>*1 = RCH or health facility*<br>*2 = Shop*<br>*3 = Family member*<br>*4 = Neighbour*<br>*5 = Radio*<br>*6= Performance by theatre group or roadshow*<br>*7 = Others*<br>*8 = Village government*<br>*8a = Newspaper*<br>*9= I don't know*	|__| heardwhere<br>otheardwhere
M8a	Where else have you heard about the discount voucher? (tick all that apply)<br>*1 = RCH or health facility*<br>*2 = Shop*<br>*3 = Family member*<br>*4 = Neighbour*<br>*5 = Radio*<br>*6= Performance by theatre group or roadshow*<br>*7 = Others*<br>*8 = Village government*<br>*8a = Newpaper*<br>*9= I don't know*	

M9	Who is eligible to receive a voucher?<br>*1=Pregnant women*<br>*2= Child < 1*<br>*3=Pregnant woman and child*<br>*4=Other (specify)*	|__| eligible<br>oteligible
M9a	Can you tell me the value of HP? *(write the amount or write 0 if doesn't know)*	|__|__|__|__|
M75	Do you ever listen to the radio?<br>*(1)Yes (2)No*<br>***If NO go to M82***	|__| femradever
M76	How frequently do you listen to the radio?<br>*1) Almost every day,*<br>*2) At least once a week,*<br>*3) less than once a week?*<br>***If (2) or (3) go to M78***	|__| femradfreq
M77	How many times a day do you listen to the radio?<br>***(Write number)***	|__| femradday
M78	At what times do you listen to the radio?<br>*1)morning 2)afternoon 3)evening 4) night*	|__| femradtime
M79	Which radio stations do you listen mostly?<br>*1) Radio free Afrika*<br>*2) Radio Tanzania (Radio ya Taifa*<br>*3) Radio Uhuru*<br>*4) Radio Clouds*<br>*5) Radio Aboud*<br>*6) Radio Tumaini*<br>*7)Radio Ukweli*<br>*8) PRT*<br>*9) Others (explain)*	|__| femradwhich
M80	**If M79==9 (others) specify**	____________________<br>femradoth
M81	Which radio programs you most like to listen?<br>*1) news bulletin 2) sports 3) films 4) Music*<br>*5) Others*	|__| femradprog
M82	Do you ever watch community drama or road shows performances?<br>*(1)Yes (2)No*<br>***If (2)No, go to M85***	|__| femdraever
M83	Last year, how many times did you watch these shows?<br>***(Write number)***	|__|__| femdranum
M84	Did you get any HATI PUNGUZO message from these performances?<br>*1)Yes 2)No*	|__| femdrahp
M85	Do you usually read newspapers?<br>*1)Yes 2)No*<br>***If 2)No, go to M87***	|__| femnews
M86	How frequently do you read newspapers?<br>*1) Almost every day,*<br>*2) At least once a week,*	|__| femnewsfreq

	3) Less than once a week?	
M87	Do you usually read posters at health facilities?<br>*1)Yes 2)No*<br>***If 2) No, go to M89***	|__| fempostusu
M88	How frequently do you read these posters?<br>*1) Almost every time when seen*<br>*2) Only once and not repeated once seen*<br>*3) Don't remember*	|__| fempostfreq
M89	In the past month, have you heard or seen any messages about HATI PUNGUZO?<br>*1)Yes 2)No*<br>***If 2)No, go to M10***	|__| femthhp
M90	*Where did you hear or see these messages?* ***(do not ask but wait for respondent to mention****) (1=Yes, 2=No)*	
	a) RADIO 1 2	femthrad
	b) NEWSPAPERS 1 2	femthnews
	c) MAGAZINES 1 2	femthmag
	d) BILLBOARDS 1 2	femthbrd
	e) FOOTBALL MATCH..1 2	femthfoot
	f) CONCERT..1 2	femthcon
	g) COMMUNITY RALLY /....................................1 2	femthral
	h) ROAD SHOW 1 2	femthroad
	i) TEE SHIRTS/CAPS 1 2	femthroad
	j) MOBILE VIDEO...1 2	femthvid
	k) TIRE PROTECTOR 1 2	femthtire
	l) CALENDAR..1 2	femthcal
	m)POSTER 1 2	femthpost
	n) STICKER 1 2	femthstick
	o) CLINIC 1 2	femthclin
	p) NYINGINE.. 1 2	femthoth

Now I would like to ask you some questions about your health right now.

M10	Have you ever been pregnant? (even if this did not lead to a live birth)<br>*1 = Yes*<br>*2 = No*	|__| everpg
M11	Are you currently pregnant?<br>*1=Yes*<br>*2=No* ***(SKIP TO M42)***	|__| currentpg
M11a	Which number pregnancy is this?<br>*(write number)*	Gravid
M12	What gestation are you now?<br>*(record no. weeks)*	|__| gestation
M13	Have you already attended the RCH this pregnancy?<br>*1 = Yes 2 = No* ***(SKIP TO M39)***	|__| attendRCH
M13a	Did you go to a clinic building for RCH services or did you go to outreach services?<br>*1= clinic building*	|__| outreach

	2= outreach	
M14	May I see your RCH card?<br>When did your first visit take place?<br>*(month/year)*<br>***Interviewer: record from card if available***	|__|__|/|__|__|__|__| firstvisit
M15	*1=Date recorded from card*<br>*2=Date reported by woman*	|__| visitverif
M16	How old was your pregnancy at the first visit? (record no.weeks)<br>***Interviewer: record from card if available***	|__|__| gestfirstvis
M17	*1=Gestation recorded from card*<br>*2=Gestation reported by woman*	|__| gestverif
M17a	Did you receive health messages from health workers during your RCH visits so far this pregnancy?<br>*1)Yes 2)No*	|__| clinicmsg
M17b	Messages were on what?<br>**(mark all mentioned)**<br>**(wait for respondent – do not prompt)**<br>*(a) Malaria*<br>*(b)Hati Punguzo*<br>*(c) HIV/AIDS*<br>*(d) Family planning*<br>*(e) Delivery of baby*<br>*(f) Looking after baby*<br>*(g)Nutrition while pregnant*<br>*(h) Dont remember*<br>*(i) Other*	clinmal|__| clinhp|__| clinhiv|__| clinfp|__| clindeliv|__| clincar|__| clinnut|__| clindk|__| clinoth|__|
M18	When you went to the clinic were you given the medicine to prevent malaria?<br>*1= Yes*<br>*2= No (Go to M21).*	
M19	Which medicine were you have given to prevent malaria? (do not prompt)<br>*1 = SP /fansidar*<br>*2= Other medicine*<br>*9= do not know*	
M21	At the clinic, were you asked if you would like to get SP medicine to prevent Malaria?<br>*1=Yes*<br>*2=No*	
M22	Why did you not use this medicine<br>*1= I don¨t like*<br>*2= It can abort the pregnancy*<br>*3= it causes other problems*<br>*4= My husband will not agree*<br>*5= Other*	
M23	Did an RCH worker give you a discount voucher for buying a mosquito net?<br>***(Interviewer: show copy of Hati Punguzo)***	|__| recdvouch

	1=Yes<br>*2=No* ***(SKIP TO M39)***	
M24	Did you have to pay money to somebody at the RCH clinic to get the voucher?<br>*1=Yes*<br>*2=No* ***(SKIP TO M26)***	|__| paidvouch
M25	How much did you have to pay? (TSh)	|__|__|__|__| paidhowmuch
M26	Was the voucher used to buy a net?<br>*1=Yes* ***(SKIP TO M29)***<br>*2=No*	|__| usevouch
M27	Why wasn't the voucher used to buy a net?<br>*1 = I gave the voucher to somebody else*<br>*2 = I already had a net*<br>*3 = I had no money to buy a net*<br>*4 = I lost the voucher*<br>*5 = I bought another commodity*<br>*6 = I sold the voucher to somebody else*<br>*7 = The place to buy a net is too far*<br>*8 = I don't know where to buy a net*<br>*9=No shop nearby selling nets*<br>*10 = Nets too expensive*<br>*11 = Other (specify).*	☐<br>☐<br>☐<br>Whynotuse<br>Otwhynotuse
M28	Do you still have the voucher?<br>*1=Yes* ***(SKIP TO M39)***<br>*2=No* ***(SKIP TO M39)***	|__| stillhave
M29	Who went to buy the net?<br>*1=Self*<br>*2=Husband*<br>*3=Relative*<br>*4=Friend*<br>*5=Other*	
M30	When was the net bought?<br>*If not known, write 1 July 2009*	|__|__|/|__|__|/|__|__|__|__| whenbuy
M31	Where was the net bought?<br>*1=Shop*<br>*2=Machinga*<br>*3=Health facility*<br>*4=Government or NGO project*<br>*5=Market*<br>*6=Other (specify)*<br>*9=Don't know*	|__| wherebuy<br>otwherebuy
M32	How long did it take you (or the person who bought the net) to get to the place where you bought the net? *(minutes) WRITE 999 IF DON'T KNOW*	|__|__|__| timebuy
M33	How much was paid as transport costs? (TSh)<br>***(Intervierwer: If paid nothing write 0; write 9999 if don't know)***	|__|__|__|__| transbuy
M34	What size is the net?<br>*1=3.5X6 2=4X6 3=6X6 4 = Other*	Sizenet<br>Otsizenet
M35	How much was paid to buy the net (after using discount voucher)?	|__|__|__|__| costnet
M36	Did you have any other difficulties in using the voucher scheme to buy a net?	|__| probbuy

	1=Yes (specify)<br>*2=No*	whatprobbuy<br>________________<br>________________<br>________________
M37	Do you still have the net that you bought with the voucher?<br>*1=yes* ***(SKIP TO M39)***<br>*2=No*	|__| stillhave
M38	If not, what happened to it?<br>*1=Stolen*<br>*2=Burnt*<br>*3=I lost*<br>*4=I sold*<br>*5=I gave it to another person*<br>*6=I sold it to another person*<br>*7 = Other (explain)*	|__| wherenet<br>otwherenet
M39	Did you sleep under a mosquito net last night?<br>*1=yes 2=No* ***(SKIP TO M42)***	|__| sleptlast
M40	Have you ever treated this net with insecticide?<br>*1 = Yes 2 = No* ***(SKIP TO M42)***	|__| evertreat
M41	When was the last time you treated the net with insecticide *(month/year)**? (If don't know write 1 July 2009)***	|__|__|/|__|__|__|__| lasttreat

Now I would like to ask you some questions about other pregnancies during the past 1 year, i.e. in 2005 and 2006 ***(Or check for well-known local event).***

M42	Did you give birth to a live child in the past year (in 2005 or 2006)?<br>*1=Yes*<br>*2=No* ***(SKIP TO C1)***	|__| livebirth
M43	How many children did you give birth to in 2005 and 2006?	|__| numbirths
M44	Did you give birth to a child that cried or showed signs of life but unfortunately died later?<br>*1=yes*<br>*2=no*	|__| childdied
M45	What is the total number of live children that you gave birth to during the past year?	|__| totlivebirth

M46 I would like to write down all the names of the children that you gave birth to in 2004 and 2005, even if they died.
(Start with the youngest one. For twins, write every child in a separate line. If there was a child was not given a name, write "not given" in the name of the child).

Number	*Name of the child (start with the youngest)*	*Born twins?<br>1 = Lone<br>2 = Twins*	*Sex<br>1=M<br>2=F*	*When was s/he born? (day/month/year)*	*Is s/he still alive?<br>1=Yes<br>2 = No*	*If s/he is still alive, how old is s/he in months*	*If the child died, when did s/he die??*	*Have you had any other children in the 2005 and 2006?<br>1=Yes 2=No*
|__|num1	Name1	|__|twins1	|__|sex1	|__|__|/|__|__|/|__|__|__|__| dob1	|__| alive1	|__|__| age1	|__|__|/|__|__|/|__|__|__|__| whendied1	|__| otherchild1
|__|num2	Name2	|__| twins2	|__| sex2	|__|__|/|__|__|/|__|__|__|__| dob2	|__| alive2	|__|__| age2	|__|__|/|__|__|/|__|__|__|__| whendied2	|__| otherchild2
|__|num3	Name3	|__| twins3	|__| sex3	|__|__|/|__|__|/|__|__|__|__| dob3	|__| alive3	|__|__| age3	|__|__|/|__|__|/|__|__|__|__| whendied3	|__| otherchild3

Ifakara Health Research and Development Centre ***in collaboration with***
Ministry of Health, Tanzania and London School of Hygiene and Tropical Medicine

Now I would like to ask you some questions about your pregnancy with each child born in 2005 and 2006.

		Name (1)<br>___________<br>*Child number* ***(from M46)***<br>Name1	*Name (2)*<br>___________<br>*Child number* ***(from M46)***<br>|__| name2	*Name (3)*<br>___________<br>*Child number* ***(from M46)***<br>|__| name3
M47	While you were pregnant with NAME, did you sleep under a mosquito net?<br>*1=Yes*<br>*2=No* ***(SKIP TO M49)***	|__| sleeppg1	|__| sleeppg2	|__| sleeppg3
M48	Had you ever treated this net with insecticide?<br>*1=yes*<br>*2=no*	|__| evertreat1	|__| evertreat2	|__| evertreat3
M49	While you were pregnant with NAME, did you attend an antenatal care clinic?<br>*1=Yes*<br>*2=No* ***(SKIP TO M75)***	|__| attendRCH1	|__| attendRCH2	|__| attendRCH3
M49a	Did you go to a clinic building for RCH services or did you go to outreach services?<br>*1= clinic building*<br>*2= outreach*	|__| outreach1	|__| outreach2	|__| outreach3
M50	How old was your pregnancy at the first visit?<br>*(weeks)*<br>***Interviewer: record from RCH card if available***	|__|__| gestfirstvis1	|__|__| gestfirstvis2	|__|__| gestfirstvis3
M51	*1=Gestation recorded from card*<br>*2=Gestation reported by mother*	|__| gestverif1	|__| gestverif2	|__| gestverif3
M52	When you went to the clinic were you given the medicine to prevent malaria?<br>*1= Yes*<br>*2= No (GO TO M55)*			
M53	Which medicine were you have given to prevent malaria? **(do not prompt)**<br>*1 = SP /fansidar*<br>*2= Other medicine*<br>*9= do not know*			
M54	When you were pregnant, how many doses did you take of that medicine?*(99 = do not know)* ***(GO TO M57)***			
M55	At the clinic, were you asked if you would like to get SP medicine to prevent Malaria? *1=Yes*<br>*2=No*			

M56	Why did you not use this medicine<br>*1= I don¨t like*<br>*2= It can abort the pregnancy*<br>*3= it causes other problems*<br>*4= My husband will not agree 5= Other (specify)*			
M57	Did an RCH worker give you a discount voucher for buying a mosquito net?<br>***(Interviewer: show copy of Hati Punguzo)***<br>*1 = Yes*<br>*2 = No* ***(SKIP TO M75)***	|__| recdvouch1	|__| recdvouch2	|__| recdvouch3
M58	Did you have to pay money to somebody at the RCH clinic to get the voucher?<br>*1=Yes*<br>*2=No* ***(SKIP TO M60)***	|__| paidvouch1	|__| paidvouch2	|__| paidvouch3
M59	How much did you have to pay?	|__|__|__|__| paidhowmuch 1	|__|__|__|__| paidhowmuch 2	|__|__|__|__| paidhowmuc h3
M60	Was the voucher used to buy a net?<br>*1=Yes* ***(SKIP TO M63)***<br>*2=No*	|__| usevouch1	|__| usevouch2	|__| usevouch3
M61	Why wasn't the voucher used to buy a net?<br>*1 = I gave the voucher to somebody else*<br>*2 =I already had a net*<br>*3 =I had no money to buy a net*<br>*4 = I lost the voucher*<br>*5 =I bought another commodity*<br>*6 =I sold the voucher to somebody else*<br>*7 = The place to buy a net is too far*<br>*8 =I don't know where to buy a net*<br>*9 = No shop nearby selling nets*<br>*10 = Nets too expensive*<br>*11 = Other (specify)*	|__| whynotuse1<br>otwhynot1	|__| whynotuse2<br>otwhynot2	|__| whynotuse3<br>otwhynot3
M62	Do you still have the voucher?<br>*1=Yes* ***(SKIP TO M75)***<br>*2=No* ***(SKIP TO M75)***	|__| havevouch1	|__| havevouch2	|__| havevouch3
M63	Who went to buy the net?<br>*1=Self*<br>*2=Husband*<br>*3=Relative*<br>*4=Friend*<br>*5=Other*			
M64	When in the course of your pregnancy was the voucher used to buy the net?<br>*1 = While pregnant*<br>*2 = after delivery*	|__| whenbuy1	|__| whenbuy2	|__| whenbuy3
M65	When was the net bought? (Month/Year)<br>***If not known, write 1 July 2009***	|__|__|/<br>|__|__|__|__| datebuy1	|__|__|/<br>|__|__|__|__| datebuy2	|__|__|/<br>|__|__|__|__| datebuy3

M66	Where was the net bought?<br>*1=Shop*<br>*2=Machinga*<br>*3=Health facility*<br>*4=Government or NGO project*<br>*5=Market*<br>*6=Other (specify)*<br>*9=Don't know*	|__| wherebuy1<br>otwherebuy1	|__| wherebuy2<br>otwherebuy2	|__| wherebuy3<br>otwherebuy3
M67	How long did it take you (or the person who bought the net) to get to the place where you bought the net? (in minutes)<br>***Write 999 if don't know***	|__|__|__| timebuy1	|__|__|__| timebuy2	|__|__|__| timebuy3
M68	How much was paid as transport costs? ***(Interviewer: if paid nothing, write 0;<br>Write 9999 if don't know)***	|__|__|__|__| transbuy1	|__|__|__|__| transbuy2	|__|__|__|__| transbuy3
M69	What size of net was bought?<br>*1=3.5X6*<br>*2=4X6*<br>*3=6X6*<br>*4=Other*	|__| sizenet1<br>otsize1	|__| sizenet2<br>otsize2	|__| sizenet3<br>otsize3
M70	How much did you pay to buy the net (after using the discount voucher)?	|__|__|__|__| costnet1	|__|__|__|__| costnet2	|__|__|__|__| costnet3
M71	Did you have any other difficulties using the voucher scheme to buy a net?<br>*1 = Yes (specify)*<br>*2 = No*	☐<br>☐<br>Probbuy1<br>Whatprob1<br>__________<br>__________<br>__________	|__| probbuy2<br>whatprob2<br>__________<br>__________<br>__________	|__| probbuy3<br>whatprob3<br>__________<br>__________<br>__________
M72	Do you still have the net that you bought with the voucher?<br>*1=yes* ***(SKIP TO M74)***<br>*2=no*	|__| havenet1	|__| havenet2	|__| havenet3
M73	If not: what happened to it?<br>*1 =Stolen*<br>*2 =Burnt*<br>*3 =I lost*<br>*4 =I sold*<br>*5 =I have given to another person*<br>*6=I sold it to another person*<br>*7 =Other (explain*<br>***(SKIP TO M75)***	|__| wherenet1<br>otwherenet1	|__| wherenet2<br>otwherenet2	|__| wherenet3<br>otwherenet3
M74	Who sleeps under the net now?<br>***(refer to line numbers from household roster H14)***	|__|__| |__|__| |__|__| |__|__| whosleeps11<br>whosleeps12<br>whosleeps13<br>whosleeps14	|__|__| |__|__| |__|__| |__|__| whosleeps21<br>whosleeps22<br>whosleeps23<br>whosleeps24	|__|__| |__|__| |__|__| |__|__| whosleeps31<br>whosleeps32<br>whosleeps33<br>whosleeps34

Module 3: Children under 1 year of age

Now I would like to ask you some questions about your infants under 1 year or any children under 1 for whom you are the guardian.

K1	Name of respondent	

		Name (1)<br>*Id of child*	*Name (2)*<br>*Id of child*
K2	Have you taken NAME to the RCH for vaccination?<br>*1=Yes*<br>*2=No*		
K3	May I see the child health card?<br>***Interviewer record:***<br>*1=Card seen*<br>*2=Card not seen*		
K4	Did you receive a Hati Punguzo insecticide retreatment kit?<br>*1=Yes*<br>*2=No*	|__|	|__|
K5	***Interviewer: is the IRK recorded on the card?***<br>*1=Yes*<br>*2=No*		
K6	How many times did you receive a kit?	☐	|__|
K7	Have you used a Hati Punguzo IRK to treat a net?<br>*1=Yes*<br>*2=No*		

Tanzania National Voucher Scheme for insecticide treated nets
HOUSEHOLD SURVEY

Ifakara Health Research and Development Centre ***in collaboration with***
Ministry of Health, Tanzania and London School of Hygiene and Tropical Medicine

Module 4: Children under 5 years of age

Interviewer: Ensure that the respondent is a mother/carer of a child under 5 years. If they have not yet given consent to be interviewed, ask for their consent.

I would like to ask you some questions about your children under 5 years or any children for whom you are the guardian

C1	Name of respondent	nameresp
C3	***Have you read him/her the consent form?***<br>1= yes<br>2=no	|__| readconsent
C4	*Does the respondent agree?*<br>*1=Yes*<br>*2=No* ***IF NO END INTERVIEW HERE***	|__| respagree

C5		*NAME (1)*<br><br>Name1	*NAME (2)*<br><br>Name2	*NAME (3)*<br><br>Name3	*NAME (4)*<br><br>Name4
C7	How old is NAME? (years)<br>*If aged less than one year, write "0"*	|__| age1	|__| age2	|__| age3	|__| age4
C8	Did NAME sleep under a mosquito net last night?<br>*1 = yes*<br>*2 = no (END)*	|__| sleepnet1	|__| sleepnet2	|__| sleepnet3	|__| sleepnet4
C9	Has the net NAME slept under ever been treated?<br>*1=Yes*<br>*2=No (END)*	|__| evertreat1	|__| evertreat2	|__| evertreat3	|__| evertreat4
C10	When was the last time the net they slept under was treated?<br>*(Month/year)* ***1 July 2009)***	|__|__|/<br>|__|__|__|__| lasttreat1	|__|__|/<br>|__|__|__|__| lasttreat2	|__|__|/<br>|__|__|__|__| lasttreat3	|__|__|/<br>|__|__|__|__| lasttreat4

Thank you very much for answering these questions.

Interviewer: Record the digital position of the household using the GPS

H24	(If no reading available, fill 99.9, 99.9, 9999)	Latitude |__|__| .<br>|__|__|__|__|__|__|__| Longitude |__|__|__| .<br>|__|__|__|__|__|__|__|
