# Supplementary material for: Vouchers for scaling up insecticide-treated nets in Tanzania: Methods for monitoring and evaluation of a national health system intervention
Source: BMC Public Health. 2008 Jun 10;8:205. doi: 10.1186/1471-2458-8-205 (PMC2442068; doi:10.1186/1471-2458-8-205)
Supplement: Additional file 2 — Facility questionnaire. [file 1471-2458-8-205-S2.pdf]

Tanzania National Voucher Scheme for insecticide treated nets
REPRODUCTIVE AND CHILD HEALTH (RCH) FACILITY SURVEY, JUNE/JULY 2007

Ifakara Health Research and Development Centre *in collaboration with*
Ministry of Health, Tanzania and London School of Hygiene and Tropical Medicine

SECTION 1: IDENTIFIERS

<table>
<tr><td></td><td>Variable Code</td><td></td><td>Variable Code</td></tr>
<tr><td>Date
|__|__| / |__|__| / |__|__|__|__|</td><td>Tarehe
Int_date</td><td>GPS Longitude
|___|___| : |___|___|___|___|___|</td><td></td></tr>
<tr><td>District
|__|__|__|</td><td>Wilaya
Distcode</td><td>GPS Latitude
|___|___| : |___|___|___|___|___|</td><td></td></tr>
<tr><td>Cluster
|__|__|__|</td><td>Cluster05
Cluster06
Cluster 07</td><td>Facility Ownership |__|
(1)Government (2)Mission (3)NGO</td><td>Facowner</td></tr>
<tr><td>Facility Type |__|
(1)Dispensary (2)Health Centre
(3)Hospital</td><td>Factype</td><td>Interviewers initials</td><td></td></tr>
<tr><td colspan="3">What is the status of the facility for the Hati Punguzo pregnant women scheme? |__|
(1)Not yet started (2)Trained but not distributing yet (3)Trained and distributing</td><td>Hpstatus_preg</td></tr>
<tr><td colspan="3">What is the status of the facility for the Hati Punguzo Infant voucher |__|
(1)Not yet started (2)Trained but not distributing yet (3)Trained and distributing</td><td>HPstatus_inf</td></tr>
<tr><td colspan="3">What is the status of the facility for the Hati Punguzo Equity voucher |__|
(1)Not yet started (2)Trained but not distributing yet (3)Trained and distributing</td><td>HPstatus_equ</td></tr>
</table>

Tanzania National Voucher Scheme for insecticide treated nets
REPRODUCTIVE AND CHILD HEALTH (RCH) FACILITY SURVEY, JUNE/JULY 2007

Ifakara Health Research and Development Centre *in collaboration with*
Ministry of Health, Tanzania and London School of Hygiene and Tropical Medicine

SECTION 2. EQUIPMENT, DRUGS AND VACCINES

Walk around the RCH with a member of staff and personally check the availability of the following:

Section 2.1 Equipment

Q2.1	Does the facility have the following equipment and materials on the day of survey?	(1)Yes (2)No	Variable Code
a.	Accessible and working adult scale?		Adultscales
b.	Accessible and working baby scale?		childscales
c.	Working watch or timing device?		Watch
d.	Supplies to mix ORS, cups and spoons		Orscup
e.	Source of clean running water (eg bucket+plug)		Water
f.	Child vaccination cards/growth monitoring cards		Vac_child
g.	Antenatal cards		Vac_mama
h.	Bed for examining pregnant women		Bed
i.	Fetalscope		Fetalscope
j.	Haemoglobin colour scale/Tallquist		Tallquist
k.	Clinsticks for testing sugar		Clinstiks
l.	Stethoscope		Stethoscope
m.	Blood pressure machine		Pressure
n.	Albusticks		Albustiks
o.	Single use needles and syringes for vaccinations		Disposable
p.	Functional sterilizer, cooker or stove		Sterilizer
q.	Functional fridge		Fridge
r.	Cold packs and cold boxes		Ice
r1	Sharps boxes		Sharp
r2	Soap		Soap
r3	Disposable gloves		Gloves
s.	TNVS vouchers for pregnant women *If no skip to 2.1t3*		Vouchers_preg
t1.	*If TNVS vouchers for pregnant women are present*:<br>How many pregnancy vouchers are there today?		Numvouch_preg
t2	*If TNVS vouchers for pregnant women are not present and the facility has launched HP:*<br>For how many days have you had no pregnancy vouchers?		Daysnohp_preg
	Questions t3, t4, t5 for all Hati Punguzo infant operating facilities:		
t3	TNVS vouchers for infants *If no skip to 2.1t5*		Vouchers_inf
t4	*If TNVS vouchers infants are present*:<br>How many infant vouchers are there today? (if more than 0 go t6)		Numvouch_inf
t5	*If TNVS vouchers for infants are not present and the facility has launched HP:*<br>For how many days have you had no infant vouchers?		Daysnohp_inf
	Questions t6, t7, t8 for HP equity voucher operating facilities:		
t6	TNVS equity vouchers (if "no" go to U)		Vouchers_equ
t7	How many equity vouchers are there today? (if more than 0 go U)		Numvouch_equ
t8	For how many days have you had no equity vouchers?		Daynohp_equ
u.	Hati Punguzo IRKits		Irkits
v.	Height stick		Stick
w.	Working electricity supply		Umeme
x.	The last time you needed emergency transport for a patient what transport did you use?<br>(1)Ambulance (2)other official vehicle (3)Public transport<br>(4)Patient's own vehicle (5)Bicycle (6)None was available		Transport

Section 2.2: Current availability of drugs
Check the drug stocks. Answer the following questions based on what you see.

Q2.2	Does the facility have the following drugs available on the day of visit?	(1)Yes (2)No	Variable Code
a.	Sulphadoxine Pyrimethamine for IPT		Spstock
b.	Vitamin A		Vitamina
c.	Ferrous/Folate		Folate
d.	Paracetamol		Paracetamol
e.	Aspirin		Aspirin
f.	Mebendazol		mebendazol
f1	Co-artemether lumifantrine		Coartem
f2	Anti-retroviral therapy (ART)		Artstock

Section 2.3: Current availability of vaccines
Check the vaccine stocks. Answer the following questions based on what you see.

Q2.3	Does the facility have the following vaccines in stock?	(1)Yes (2)No	Variable Code
g.	BCG vaccine		Bcg
h.	OPV vaccine		Opv
i.	DPT vaccine		Dpt
j.	Measles vaccine		Measles
k.	TT vaccine		Tetanus
l.	Were *any* (polio) vaccines indicated as unusable by the Visual Vaccine Monitor?		Vvm
m	Which staff member assisted in this section?		

Section 2.4: Current availability of diagnostics

Q2.4	Which of the following diagnostic methods are available in this clinic for malaria?		Variable Code
n	Clinical diagnosis available		Clinical_mal
o	Rapid test for malaria in stock today		Rapid_mal
p	Functioning microscopy (slides plus microscope plus slide reader)		Fmicro_mal
q	Non-functioning microscopy		Nonmicro_mal
r	No method of diagnosing malaria		Nomethod_mal
s	Does the facility offer HIV diagnostics?<br>(1)Yes at this clinic (2)Yes at another facility/laboratory (3)No<br>*If yes (1) go to t. If yes (2) go to W. If no (3) go to Section 3*		Diagnose_HIV
	If yes, clinic offers HIV diagnostic facility on site:<br>Do you have the following rapid antibody tests available in clinic today?		
t	Capillis (1 yes/ 2 no)		Capillis
u	SD Bioline (1 yes/ 2 no)		SD Bioline
v	Determine (1 yes/ 2 no)		Determine
w	How many days does it take to get the results?		HIV_days

	If results available on same day write '0'.		

SECTION 3 FACILITY SERVICES

Discuss with the head of facility to determine which services are routinely offered and the health workers who usually have responsibility for specific tasks.

Section 3.1 Services available

Q3.1			Variable Code
a.	How many days per week is the facility open?		Ser_days
	What services are routinely offered at this clinic?	(1)yes (2)no	
b.	Antenatal registration and counselling		Ser_reg
c.	Vaccination		Ser_vac
d.	VCT for pregnant women		Ser_vct
d1	PMTCT (counselling and testing, ARV prophlaxis for mother and newborn, infant feeding and family planning counselling)		Ser_pmtct
e.	Family planning		Ser_fp
f.	Child health		Ser_child
f1	Active outpatient clinic (OPD)		Ser_OPD
f2	Post-natal care		Ser_postnatal
g.	How many days per week are antenatal health services provided? (write number of days)		Ser_ancdays
	Note: deleted out g1-g5 - days of week antenatal provided		
h.	How many days per week are health education services provided? (write number of days)		Ser_hedays
h1	How many days per week are child health services provided? (write number of days)		Ser_infdays
	**If Trained and distributing HP for pregnant women: (all)**		
j.	What was the date when the first HP pregnancy voucher was issued in this clinic? (dd/mm/yy): write 99 if don't know dd	|__|__|/|__|__|/|__|__|	Faclnch_preg
	**If trained and distributing HP for infants:**		
j1.	What was the date when the first HP infant voucher was issued in this clinic? (dd/mm/yy): write 99 if don't know dd	|__|__|/|__|__|/|__|__|	Faclnch_inf
	**If trained and distributing HP equity voucher:**		
j2.	What was the date when the HP equity voucher was issued in this clinic? (dd/mm/yy): write 99 if don't know dd	|__|__|/|__|__|/|__|__|	Faclnch_equ
k.	Does this clinic offer outreach services?<br>(1)Yes for pregnant women only<br>(2)Yes for children only<br>(3)Yes, for both pregnant women and children<br>(4)No *If no skip to S. 3.2*		Outreach
	Questions 3.1K1, L, 3.1L1 and 3.1L2 deleted		
m.	During the last outreach service did you offer the following services? (1)Yes (2)No		
n.	Distribute IPT		Out_ipt
o.	Distribute Hati Punguzo for pregnant women		Out_hppreg
o1	Distribute Hati Punguzo for infants		Out_hpinf
o2	Distribute Hati Punguzo equity voucher		Out_hpequ
	Delete 2006 questions s31p, q, r		
s.	Why would you not give a pregnant woman at outreach Hati Punguzo?<br>(1)she can't afford to use the voucher (2)she lives too far from a shop (3)she doesn't want a voucher (4)not enough vouchers in clinic (5)no book to take on outreach (6)other (specify)______________________ (7)all receive		Notgivehp_preg

Section 3.2 Characteristics of health workers by responsibility

Ask the in-charge to tell you the following about the staff at the RCH.

Q3.2		Prescriber	Nurse	RCH Aide	Medical Attendant	Record er	VHW	Other (sp.)	Total
a.	No. in RCH facility	S32a1	S32a2	S32a3	S32a4	S32a5	S32a 6	S32a7	S32a8
b.	No. providing antenatal care	delete							
c.	No. doing health education	delete							
d.	No. providing VCT services								
e.	No. doing registrations	delete							
f.	No. providing f/p services	delete							
g.	No. providing child services	delete							
h.	No. trained in HP- preg								
h1	No trained in HP- child								
h2	No. trained in HP- equity								
i.	How many are working today?								
j.	How many are away on training today?								
J1.	How many off sick today?								
J2.	How many on leave today?								
J3	How many away on official clinic work?								
J4	How many own a mobile phone?								
J5	How many have a mobile phone with them today?								
k	Staff member who assisted								

Tanzania National Voucher Scheme for insecticide treated nets
REPRODUCTIVE AND CHILD HEALTH (RCH) FACILITY SURVEY, JUNE/JULY 2007

Ifakara Health Research and Development Centre *in collaboration with*
Ministry of Health, Tanzania and London School of Hygiene and Tropical Medicine

Section 3.3 Supervision

Ask the In-Charge about supervision visits received over the last six months, plus details about the last visit. If respondent is unsure try looking in the visitors book

Q 3.3			Variable Code
a.	Where you present at the last supervision visit?<br>(1)Yes (2)No		Lastsup
b.	How many times during the last six months did the facility receive a supervisory visit? *(write number)*	If 0 skip to Sect 4.	Numsup
	Now please think about the last supervision visit.<br>Which supervisors came on the last visit?	(1)Yes<br>(2)No	
c.	DMO		Sup_dmo
d.	Other Medical doctor		Sup_dr
e.	RCH co-ordinator		Sup_rch
f.	Vaccine official		Sup_inj
g.	Mfamasia		Sup_pharm
h.	Other (specify)__________		Sup_other
	During the last supervision visit did your supervisors spend time with any service providers to discuss:	(1)Yes<br>(2)No<br>(3)DK	
i.	Family planning services		fp_sup
j.	Vaccinations		vac_sup
k.	Health education		he_sup
l.	Physical examination of antenatal women		mama_sup
m.	Physical examination of children		child_sup
n.	VCT		vct_sup
o.	Hati Punguzo		hp_sup
p.	Staff member who assisted?		

<u>SECTION 4 FACILITY RECORDS MODULE</u>

Section 4.1 Ledger (Book 4) – delete 4.1

Section 4.3 MTUHA (Book 2)

Ask to see MTUHA book 2 with records from July 2006 to June 2007. Look for Table 27A and Table 25A. You need to record the number of children under 5 years who were diagnosed with malaria, and total attendees under 5, for each month. If records are not available for any month enter -1.

Notice here that 2006 these questions ran from s43a to s43af. These are not asked again in 2007.
Note this year the questions run from s43ag to are for period July2006-June 2007.

ag.	2006 JULY	Malaria cases, children<5	S43ag	Attendees, children<5	S43ag1
ah.	2006 AUGUST	Malaria cases, children<5		Attendees, children<5	
ai.	2006 SEPTEMBER	Malaria cases, children<5		Attendees, children<5	
aj.	2006 OCTOBER	Malaria cases, children<5		Attendees, children<5	
ak.	2006 NOVEMBER	Malaria cases, children<5		Attendees, children<5	
al.	2006 DECEMBER	Malaria cases, children<5		Attendees, children<5	
am.	2007 JANUARY	Malaria cases, children<5		Attendees, children<5	
an.	2007 FEBRUARY	Malaria cases, children<5		Attendees, children<5	
ao.	2007 MARCH	Malaria cases, children<5		Attendees, children<5	
ap.	2007 APRIL	Malaria cases, children<5		Attendees, children<5	
aq.	2007 MAY	Malaria cases, children<5		Attendees, children<5	
ar.	2007 JUNE	Malaria cases, children<5		Attendees, children<5	

Section 4.2 Antenatal attendees and Hati Punguzo vouchers: MTUHA (Book 6)

Ask the health worker assisting you to let you see all the MTUHA book 6 available in the clinic. In some clinics there are separate books for different villages. You first need to find out how many MTUHA book 6 there are. Do they cover the period March to June 2007? Now tell the PDA how many books there are. The PDA will then ask you the following questions FOR EACH of the books separately.

		<20 weeks	>20 weeks	Variable Code
Q4.2	**In June:**			
a.	What is the total number of visits to the facility for antenatal?	S42a1	S42a2	
b.	How many of these antenatal attendees received a voucher?	S42b1	S42b2	
c.	How many of these antenatal attendees received IPTp 1?	S42c1	S42c2	
	d and e deleted for 2007			
	**In May:**			
f.	What is the total number of visits to the facility for antenatal?			
g.	How many of these antenatal attendees received a voucher?			
h.	How many of these antenatal attendees received IPTp 1?			
	i and j deleted for 2007			
	**In April:**			
k.	What is the total number of visits to the facility for antenatal?			
l.	How many of these antenatal attendees received a voucher?			
m.	How many of these antenatal attendees received IPTp 1?			
	n and o deleted for 2007			
	**In March:**			
p.	What is the total number of visits to the facility for antenatal?			
q.	How many of these antenatal attendees received a voucher?			
r.	How many of these antenatal attendees received IPTp1?			
	s and t deleted for 2007			
u.	Which member of staff assisted?			
v.	*Ask the member of staff who gives out Hati Punguzo*<br>Why do you sometimes not give a pregnant woman a voucher? *(write response)*<br>(1)She still can't afford to buy a net (2)She lives too far from the shops (3)She does not need it because she already has a net (4)Other (specify)____________________(5)all are given			*notgivehp_cli*

Section 4.4 Hati Punguzo equity voucher

Ask to see the Hati Punguzo equity voucher register for pregnant women and carers of infants who have received a equity voucher since March 2007.

a.	How many equity vouchers were given out in **June**?		Equjune
b	How many pregnant women received an equity voucher in June?		Equjune_preg
c	How many carers of infants received an equity voucher in June?		Equjune_inf
d	How many equity vouchers were given out in **May**?		Equmay
e	How many pregnant women received an equity voucher in May?		Equmay_preg
f	How many carers of infants received an equity voucher in May?		Equmay_inf
g.	How many equity vouchers were given out in **April**?		Equapr
h	How many pregnant women received an equity voucher in April?		Equapr_preg
i	How many carers of infants received an equity voucher in April?		Equapr_ing
j.	How many equity vouchers were given out in **March**?		Equmar
k	How many pregnant women received an equity voucher in March?		Equmar_preg
l	How many carers of infants received an equity voucher in March?		Equmay_inf
m	What is the most difficult thing about the equity voucher? (specify)<br>____		Problem_equ

Section 4.5 Infants attending for measles and Hati Punguzo for infants: MTUHA (Book 6)
Ask the health worker assisting you to let you see all the MTUHA book 6 available in the clinic where measles vaccinations and Hati Punguzo for infants is recorded for the last 4 months. In some clinics there are separate books for different villages. You first need to find out how many MTUHA book 6 there are. Do they cover the period March to June 2007? Now tell the PDA how many books there are. The PDA will then ask you the following questions FOR EACH.

			Variable Code
Q4.5	**In June:**		
a.	What is the total number of infant visits to the health facility for measles vaccinations?		Infjune
b.	How many of these infants received an infant voucher?		Infjune_hp
	**In May:**		
c.	What is the total number of infant visits to the health facility for measles vaccinations?		Infmay
d.	How many of these infants received an infant voucher?		Infmay_hp
	**In April:**		
e.	What is the total number of infant visits to the health facility for measles vaccinations?		Infapr
f.	How many of these infants received an infant voucher?		Infapr_hp
	**In March:**		
g.	What is the total number of infant visits to the health facility for measles vaccinations?		Infmar
h.	How many of these infants received an infant voucher?		Infmar_hp
i.	Why would you not give an eligible child Hati Punguzo for infants? (1)the carer can't afford to use the voucher (2)the family lives too far from a shop (3)the carer doesn't want a voucher (4)not enough vouchers in clinic (5)no book for infant vouchers on outreach (6)all eligible children always get a voucher (7)other (specify)____________________		
j.	What is the most difficult thing about the infant voucher? (specify) ____		Problem _inf

SECTION 5. HEALTH EDUCATION/PROMOTION
Section 5.1 Observation of health promotion materials on display

		(1)Yes (2)No	Variable Code
Q5.1	On the day of survey were there posters displayed which addressed the following:		
a.	Hati Punguzo		Post_hppreg
b.	Ngao		Post_ngao
c.	IPT – SP		Post_IPT
d.	STI		Post_sti
e.	Nutrition		Post_nut
f.	HIV		Post_HIV
g.	Family Planning		Post-fp
h.	Childhood illnesses (measles, polio, neonatal tetanus)		Post_child

Section 5.2: Observation of key messages delivered during health education sessions either with pregnant women or carers of children under 5 or mixed sessions.
Ask for permission to observe a health education session – either group or individual. If possible select to observe a group session. For each topic below write down whether it was discussed.

Q5.2			Variable Code
a.	What type of health education session was observed?<br>(1)Group (2)Individual (3)Did not observe	*If (1) go to b.<br>If (2) go to c.<br>If (3) go to e.*	He_observed
b.	Were the attendees of the group session<br>(1)Pregnant women only (2)Mothers of children <5yrs only (3)Mixed	*Now go to f.*	He_attendees
c.	In the individual session observed:<br>What number visit to the RCH was it for the pregnant woman?		S52c
d.	In the individual session observed:<br>Was it the woman's first pregnancy?	*Go to F*	He_firstpreg
e.	Why did you not observe a health education session? (Specify)<br>____	*Go to 5.3*	S52e
	Where the following topics discussed in the health education session:	(1)Yes<br>(2)No	
f.	Aims and Importance of Attendance at RCH<br>(eg services available, timing of visits)		He_aims
g.	Individual birth plan<br>(eg where to deliver, emergencies, finances, transport)		He_birthplan
h.	Breastfeeding		He_feeding
i.	Nutrition<br>(eg importance in pregnancy, what foods to eat, for the infant)		He_nutrition
j.	Malaria<br>(eg causes, consequences, treatment, ITNs and IPT)		He_malaria
k.	HIV<br>(eg risk factors, consequences, VCT, ARV)		He_hiv
l.	Anaemia<br>(causes, consequences, detection, treatment)		He_anaemia
m.	Was a specific mention made of Hati Punguzo pregnant vouchers?		He_hppreg
m1	Was a specific mention made of Hati Punguzo infant vouchers?		He_hpinf
m2	Was a specific mention made of Hati Punguzo equity vouchers?		He_hpequ
n.	Was there an explanation of who Hati Punguzo was meant for and who could get one?		He_hpelig
o.	Was there an explanation of the value of Hati Punguzo?		He_hpvalue
p.	Were the pregnant women/carers of children told which shops they could use Hati Punguzo to buy a bednet in their area?		He_hpshops

Section 6 Observation of actual delivery of interventions – delete, keep only Q6c, total number of pregnant women visits on day of survey

Q6	At which point were the following given to the pregnant woman?<br>(1)Registration (2)Counselling (3)Health education (4)Other (5)Not			Variable Code
a.	IPT			Dist_ipt
a.1	If IPT was 'Other' specify:<br>--			
b.	Hati Punguzo for pregnant women			Dist_hp
b.1	If Hati Punguzo was 'Other' specify:<br>--			
c.	What was the total number of visits to the health facility for antenatal services by the end of the day of survey?	<20 wks	>20 wks	
