# Supplementary material for: Vouchers for scaling up insecticide-treated nets in Tanzania: Methods for monitoring and evaluation of a national health system intervention
Source: BMC Public Health. 2008 Jun 10;8:205. doi: 10.1186/1471-2458-8-205 (PMC2442068; doi:10.1186/1471-2458-8-205)
Supplement: Additional file 3 — Facility users survey questionnaire. [file 1471-2458-8-205-S3.pdf]

Tanzania National Voucher Scheme for insecticide-treated nets
EXIT SURVEY, JUNE/JULY 2007

Ifakara Health Research and Development Centre *in collaboration with*
Ministry of Health, Tanzania and London School of Hygiene and Tropical Medicine

SECTION 1: IDENTIFIERS

	Variable Code		Variable Code
Date<br>|__|__| / |__|__| / |__|__|__|__|	Int_date	District |__|__|__|	Wilaya<br>Distcode
Ward/Cluster |__|__|__|	Cluster 05<br>Cluster 06<br>Cluster 07	Kitongoji |__|__|__|	
Facility Code |__|__|__|	facilityco	Facility Type |__|	typefac
Interviewer ID |__|__|		Respondent ID |__|__|__|	
Hati Punguzo ya wajawazito?	Hati Punguzo Maalum?	Hati Punguzo ya Watoto?	

SECTION 2: SOCIO ECONOMIC BACKGROUND OF RESPONDENT

Explain to the respondent that the first questions are about her background.

Q2			Variable Code
a.	What is your birth date?<br>*(dd/mm/yyyy) (if don't know 01/07/2099)*	|___|___|/|___|___|/|___|___|___|	S2a
b.	What is your age now?<br>(write years)		S2b<br>Age4
c.	How many years at school have you completed?<br>(write number of years)		Educ<br>Educgrp<br>Educ3
d.	Have you ever been married?<br>(1)Yes, currently married (2)Yes but not anymore (3)Living with partner but not married (4)Never		Marstat
	How many people live in your household? *(including yourself)*		
e.	Adults > 18 years		S2e
f.	Children 5-17 years		S2f
g.	Children <5 years		S2g
h.	Which District do you live in?<br>(1)this District (2)other District		disresid
i.	Which Region do you currently live in?<br>(1)this Region (2)other Region		regresid
i1	Do you rent this house?<br>*(1) yes (2) no (3)=Other (specify)*		Renthouse
j.	Does the household you live in have a cement floor?<br>(1)Yes (2)No		Cement
k.	Does the household you live in have a tin/tiled roof?<br>(1)Yes (2)No		Roof
l.	Does your household have an electricity supply?<br>(1)Yes (2)No		Umeme
m.	Is there a landline telephone in your household?<br>(1)Yes (2)No		Phone
n.	What kind of toilet facilities does your household have?		Toilet

	(1)Flush (2)Pit/latrine (3)No facility/bush/field (4)Other		
	Does anyone living in your household own a:<br>(1)Yes (2)No		
o.	Radio		Radio
p.	Television		TV
q.	Bicycle		Bike
r.	Pikipiki		Piki
s.	Car/truck		Car
t.	Mobile phone		Mobile
u.	Bednet	*If NO, skip to S31a*	Bednet
v.	How many bednets are there altogether?<br>(write number)		Numnets
w.	How many of these bednets have ever been treated with insecticide?		Numever

SECTION 3.1: PREGNANCY HISTORY
Explain to respondent that you would now like to ask some questions about her pregnancy.

Q3.1			Variable Code
a.	What is your current gestation?<br>(in weeks)		gestoday
b.	Which number pregnancy is this?<br>(write number)		S31b
c.	In total how many live births have you had?<br>(write number)	*If 0 skip to Sect. 3.2*	primi
d.	What was the birth date of your last born child? dd/mm/yyyy<br>(if don't know: 01/07/2009)	|__|__| / |__|__| / |__|__|__|__|	

SECTION 3.2 THIS PREGNANCY
Ask to see clinic card to verify information

Q3.2			Variable Code
a.	How many times have you attended the RCH for antenatal services so far this pregnancy (including today)?		S32a<br>Visit4
b.	What was your gestation at the first visit?<br>*(write number of weeks)*		Gest1stvisit
c.	(If > 1 visit): What was your gestation at the second visit?		Gest2ndvisit
d.	(If > 2 visits): What was your gestation at the third visit?		Gest3rdvisit
e.	(If > 3 visits): What was your gestation at the fourth visit?		Gest4thvisit
f.	Have you ever been given iron prophylaxis from the RCH?<br>(1)Yes (2)No		Getiron
f1.	At which visit were you given iron prophylaxis?<br>(1)1st (2)2nd (3)3rd (4)4th		Ironvisit
g.	Have you ever been given IPTp1 (first dose) from the RCH?<br>(1)Yes (2)No		Getipt1

g1.	At which visit were you given IPTp1 (first dose)? (1)1st (2)2nd (3)3rd (4)4th		Ipt1visit
h.	Have you ever been given IPTp2 (second dose) from the RCH? (1)Yes (2)No		Getipt2
h1.	At which visit were you given IPTp2 (second dose)? (1)1st (2)2nd (3)3rd (4)4th		Ipt2visit
i1	Were you asked if you wanted VCT?		AskVCT
i.	Have you ever been given ARV? (1)Yes (2)No		Getarv
j.	Have you ever been given TT vaccine from the RCH? (1)Yes (2)No		Gettt
k.	Have you been given a HP pregnancy voucher from the RCH during this pregnancy? (1) Yes (2)No		S32k
k1.	At which visit were you given a voucher? (1)1st (2)2nd (3)3rd (4)4th		S32k1
K2	Have you ever received an equity voucher? (1)yes (2)no *(if no)*		
K3	*(If K2 NO)* Do you think you will receive an equity voucher? (1)Y (2)N (3)DK		
l.	After how many weeks should you visit the clinic again? (1)1-2 (2)3-4 (3)5-6 (4)7-8 (5)9-10 (6)10+ (7)don't know		nextvisit

SECTION 4: VOUCHER KNOWLEDGE, USE AND ITN OWNERSHIP

Remind the respondent that all the information she provides is very valuable to try to improve services and that her name or address is not written anywhere so whatever she tells us she can never be identified. Ask her to answer as honestly as she can.

Section 4.1 ITN use

Q4.1		Variable Code	
aa	Before you were pregnant did you normally sleep under a bednet? (1)Yes (2)No		Beforepreg _any
a.	Have you ever slept under a bednet during this pregnancy? (1)Yes (2)No	*If no skip to g.*	Usenet
b.	Was it a treated net? (1)Yes (2)No (3)Don't know		Useitn
c.	This pregnancy, during which month of gestation did you first sleep under a bednet? (write month 1-9)		Pregest _net
d.	Did you sleep under a bednet last night? (1)Yes (2)No	*If no skip to g.*	Netlast
d1	What type of net was it? Ordinary net? Olyset net? Permanet? Don't know		Typenet
e.	Did you ever treat this net with insecticide? (1)Yes (2)No	*If no skip to g.*	ITNlast
f.	When was the last time this net was treated with insecticide? (write day/month/year)		S41f Treatlast12
f1	What was the type of insecticide put on the net? (1)Ngao (2)KO123 (LLasting) (3)Other (specify__________) (4)Don't know		Insecticide
f2	After how many months should you put insecticide again?		retreat
g.	Who else normally shares your bed?		S41g1-

	(1)Child<5yrs (2)Child>5yrs (3)Husband (4)No-one (5)Other		S41g5

Section 4.2 Hati Punguzo

Q4.2		Variable Code	
aa.	Have you heard of the Hati Punguzo? The discount voucher programme for pregnant women to buy a bednet at a cheaper price?<br>(1)Yes (2)No		Heardhp_<br>preg
Ab	If yes, where did you first hear about HP for pregnant women?<br>1 = RCH or health facility, 2 = Shop 3 = Family member<br>4 = Neighbour, 5 = Radio, 6= Performance by theatre group or roadshow, 7 = Others, 8 = Village government, 8a = Newpaper<br>9= I don't know		Firstheard_<br>preg
Ac	Where else have you heard about HP for pregnant women? (tick all that apply)<br>*1 = RCH or health facility, 2 = Shop, 3 = Family member*<br>*4 = Neighbour, 5 = Radio, 6= Performance by theatre group or roadshow, 7 = Others, 8 = Village government, 8a = Newpaper*<br>*9= I don't know*		Allheard_rc<br>hpreg<br>Allheard_s<br>hoppreg<br>ect
Ad	Can you tell me what the value of the voucher for pregnant omen is?<br>(enter the amount in numbers or enter 0 is doesn't know)		
Ae	Who is eligible to receive a HP voucher for pregnant women?<br>(1)pregnant woman (2)child under 1 year (3)Pregnant woman and child under 1 (4)other (specify)		
Ba	Have you heard of the Hati Punguzo for infants? The discount voucher programme for infants to buy a bednet at a cheaper price?<br>(1)Yes (2)No		
Bb	If yes, where did you first hear about HP for infants?<br>1 = RCH or health facility, 2 = Shop 3 = Family member<br>4 = Neighbour, 5 = Radio, 6= Performance by theatre group or roadshow, 7 = Others, 8 = Village government, 8a = Newpaper<br>9= I don't know		
Bc	Where else have you heard about HP for infants? (tick all that apply)<br>*1 = RCH or health facility, 2 = Shop, 3 = Family member*<br>*4 = Neighbour, 5 = Radio, 6= Performance by theatre group or roadshow, 7 = Others, 8 = Village government, 8a = Newpaper*<br>*9= I don't know*		
Bd	Can you tell me what the value of the voucher for infants is?<br>(enter the amount in numbers or enter 0 is doesn't know)		
Be	Who is eligible to receive a HP voucher for infants?<br>(1)pregnant woman (2)child under 1 year (3)Pregnant woman and child under 1 (4)other (specify)		
Ca	Have you heard of the Hati Punguzo equity voucher?<br>(1)Yes (2)No		
cb	If yes, where did you first hear about HP equity voucher?<br>1 = RCH or health facility, 2 = Shop 3 = Family member<br>4 = Neighbour, 5 = Radio, 6= Performance by theatre group or roadshow, 7 = Others, 8 = Village government, 8a = Newpaper<br>9=cd I don't know		
Cc	Where else have you heard about HP equito voucher (tick all that apply)<br>*1 = RCH or health facility, 2 = Shop, 3 = Family member*<br>*4 = Neighbour, 5 = Radio, 6= Performance by theatre group or roadshow, 7 = Others, 8 = Village government, 8a = Newpaper*<br>*9= I don't know*		
Cd	Can you tell me what the value of the HP equity voucher is?		

	(enter the amount in numbers or enter 0 is doesn't know)		
Ce	Who is eligible to receive a HP equito voucher?<br>(1)pregnant woman (2)child under 1 year (3)Pregnant woman and child under 1 (4)other (specify)		
i.	This pregnancy have you been given Hati Punguzo for pregnant women voucher from the RCH? (show a voucher)<br>(1)Yes (skip to k) (2)No		Gethp
j.	If No:<br>Did you want to be given a pregnant women voucher?<br>(1)Yes (2)No (go to Y)		Wanthp
j1	If Q42j=yes:<br>Why do you think you were not given a voucher? (record response)<br>____ *Now skip to Y*		
k.	If did receive a voucher from RCH:<br>On which visit this pregnancy did you receive the voucher?<br>(1)First (2)Second (3)Third (4)Fourth (5)Fifth		HPvisit
l.	Did you pay anyone some money to get it?<br>(1)Yes (2)No (skip to n)	*If no skip t n*	Paidhp
m.	If yes:<br>How much money did you pay for the voucher? (Tsh)		
n.	Was the Hati punguzo pregnancy voucher used yet to buy a bednet?<br>(1)Yes (2)No (skip to 4.2 t)	*If no skip to t*	Usedhp
n1	*Was the Hati punguzo maalum voucher used to buy a bednet?*		*Usedhp_equ*
o.	What size net was bought with the voucher?<br>(1) 3.5X6 (2) 4X6 (3) 6X6 (4)Other		Sizehpnet
p.	What was the amount of money that had to be added to the Hati Punguzo to buy the net? (write the amount in numbers)		Topup
q.	When was the net bought using the Hati Punguzo voucher?<br>(day/mth/yr (DK: 01/07/2009)		Datehpnet
r.	Who now uses the bednet bought with the voucher?<br>(1)Myself (2)My husband (3)My child (4)Another relative living with me (5)Adult outside my house (6)Child outside my house		Usehpnet
s.	How easy was it to use would you say:<br>(1)Very easy (2)OK (3)Not so easy (4)Very difficult	*Now skip to Y*	
	If not yet used:		
t.	Do you still have the voucher?<br>(1)Yes (skip to W) (2)No	*If yes skip to w.*	Stillhp
u.	*If no:*<br>What happened to it?<br>(1)Stolen (2)Burnt (3)Lost it (4)Sold it (5)Gave it away (6)Other		
v.	*If Other explain*___________________________________	*Now skip to Y*	
w.	*If yes, still has the voucher:*<br>Do you plan to use the voucher to buy a net for someone?<br>(1)Yes for myself (2)Yes for my family (3)Yes for someone else (4)No	*If Yes (1-3) skip to Y*	Plantouse
x.	If doesn't plan to use: Why don't you plan to use the voucher?<br>(1)Already have a net (2)Don't like bednets (3)No money (4)Other (specify)________________________		
y	This pregnancy have you received the HP equity voucher?<br>(1)Yes (go to X) (2)No		Gethp_equ
Y1	*If no:*<br>Do you want to receive HP equity voucher?<br>(1)yes (2)no (go to Sec 5)		Wanthp_equ
Y2	If 42y1=(1)yes<br>Why do you think you did not receive HP equity voucher?		

	___________________________(specify) (now go to Sec 5)		
Z	*If did receive HP equity voucher:*<br>Did you have to pay for the equity voucher?<br>(1) Yes (2)No		Paidhp_equ
Z1	How much did you pay?		
Z2	Have you already used the HP equity voucher?<br>(1) Yes (go to Sec 5) (2) No		
Z3	*If no, not yet used:*<br>Why have you not yet used the HP equity voucher?<br>___________________________ specify		

SECTION 5. KEY BEDNET RETREATMENT INDICATORS

Ask the respondent the following questions. Do not prompt her with the coded answers – rather wait to see what responses she gives.

S 5		Variable Code	
5.1	Have you heard of insecticide for bednets?<br>(1)yes (2)no		**Heardnets**
5.2a	Have you heard of Ngao for bednets?<br>(1)yes (2)no		**Heardngao**
b	If yes:<br>If yes, where have you heard about Ngao for bednets?<br>1 = RCH or health facility, 2 = Shop 3 = Family member<br>4 = Neighbour, 5 = Radio, 6= Performance by theatre group or roadshow, 7 = Others, 8 = Village government, 8a = Newpaper<br>9=cd I don't know		**Whereheard_ngao**
c	How often do you have to treat your bednet with Ngao? (write months)		**Retreat_ngao**
d	How much does Ngao cost? (write Tsh)<br>(99 if don't know)		**Cost_ngao**
e	Have you ever used Ngao to treat a bednet<br>(1)yes (2)no		**Everused_ngao**
f	If yes:<br>How many months ago did you use?		**Monthsago_ngao**
	If yes: What was inside the packet?		
G1	Instructions		**Instructions_ngao**
G2	Gloves		**Gloves_ngao**
G3	Dawa		**Dawa_ngao**
G4	Binder		**Binder_ngao**
5.3a	Have you ever heard of Ngao ya muda mrefu (long lasting net treatment)?<br>(1) yes (2)no		**heardngaoll**
b	If yes:<br>If yes, where have you heard about Ngao LL for bednets?<br>1 = RCH or health facility, 2 = Shop 3 = Family member<br>4 = Neighbour, 5 = Radio, 6= Performance by theatre group or roadshow, 7 = Others, 8 = Village government, 8a = Newpaper<br>9=cd I don't know		
c	How often do you have to treat your bednet with Ngao LL? (write months, 99 if doesn't know; 60 if never again)		
d	How much does Ngao LL cost? (write Tsh)<br>(99 if don't know)		
e	Have you ever used Ngao LL to treat a bednet<br>(1)yes (2)no		
f	If yes: How many months ago did you use?		
	If yes: What was inside the packet?		

G1	Instructions		
G2	Gloves		
G3	Dawa		
G4	Binder		
5.4	*If heard of Ngao LL:*<br>Can you tell me what the difference is between Ngao and Ngao LL?<br>_________________________ (specify)		

Thank the respondent for taking the time to take part in the survey.
