# Supplementary material for: Vouchers for scaling up insecticide-treated nets in Tanzania: Methods for monitoring and evaluation of a national health system intervention
Source: BMC Public Health. 2008 Jun 10;8:205. doi: 10.1186/1471-2458-8-205 (PMC2442068; doi:10.1186/1471-2458-8-205)
Supplement: Additional file 4 — Community group interview topic guide. [file 1471-2458-8-205-S4.pdf]

Ifakara Health Research and Development Centre and London School of Hygiene and Tropical Medicine

Monitoring and Evaluation of the Tanzania National Voucher Scheme for insecticide-treated nets

Community Group Interview Topic Guide: Women who are pregnant &/or have young babies

After a general introduction about the reasons for convening the group then move onto:

General Antenatal Issues
Discuss the steps the women think are important in helping to maintain the health of themselves and their baby during pregnancy?

Ask the women to tell the story of their last pregnancy. Probe for: How many visited an antenatal clinic at least once during this (or your last) pregnancy?

> For those who said they have used a clinic probe for: how often they had attended the ANC; how far into the pregnancy they made their first visit and why did they make the visit; who was involved in the decision to visit the clinic; their perceptions about how many times a woman should visit a clinic during her pregnancy and why; the factors that influence actual clinic attendance; the services they expected to receive at the ANC, if they received them and if not then their perceptions of why these services weren't offered; how much it costs to attend the ANC (transport, drugs, tests etc) and where does the money come from; any problems they encountered during their visits to the ANC and their perceptions of why these problems occurred.
>
> For those who said they hadn't used a clinic probe for: their reasons for not visiting and who was involved in the decisions not to visit the ANC

Discuss with the whole group their perceptions of what factors influence women to use antenatal clinics; the problems they have in accessing the ANC; if they know of women who never use the ANC services and their perceptions of why this might be the case.

General Hati Punguzo
Ask the women if they have heard of the Hati Punguzo programme.
For those that say they have heard: ask them to describe what the programme is. For those that haven't: ask if they've heard about the vouchers that are available at the antenatal clinics for pregnant women

Probe to find out: where the women had heard about the scheme; what they had heard; their perceptions about who should be given a voucher, what can they be used for, where they can be used them and how much are they worth.

Ask the women to describe their experiences of accessing the vouchers at antenatal clinics. Probe to find out: who was offered a voucher; when the voucher was offered (on first visit or subsequent visits?); whether or not they took the voucher if it was offered.

If there are women who hadn't been given a voucher then ask them to discuss the reasons why they think they weren't offered a voucher. Probe for: whether or not they expected to be offered a voucher (and if not then why not?); if they had expected to be offered a voucher and weren't then what are their perceptions of why they weren't offered one and what did they do when one wasn't offered?

For those that received a voucher, ask whether or not they had redeemed the voucher.

Ask the women who had redeemed their voucher to tell the story of the process.

Probe for: length of time they kept the voucher before redemption; those involved in the decision about whether to redeem the voucher or not and when it should be redeemed; any problems in getting a net using the voucher; whether or not the net came with Ngao [insecticide sachet] and if they used it to treat the net; which members of the family are sleeping under the voucher net and why these people are the ones who are using the net.

Ask the women who had received a voucher but not redeemed it the reasons for their non-redemption:

Probe for: how long the voucher had been in their possession; who was involved in the decision not to use the voucher; the factors that prevented them from using the voucher and whether or not they were currently sleeping under a net.

Discuss with the women their perceptions about how much money are they are supposed to add to get a net when using Hati Punguzo and from where they had received information about the amount they were supposed to ‘top-up’ to get a net. Probe for: their perceptions about what determines the amount of top-up they have to pay (e.g. whether or not the amount of top-up is related to net size, or any other factors) and whether or not the amount of top-up they had to pay was different from their expectations.

Ask the women to discuss whether or not they think that the Hati Punguzo programme has made any difference to women’s use of the antenatal clinics (probe for: whether or not the programme has had an impact on the number of women who use antenatal clinics during their pregnancies, the timing of their first visit, and/or the frequency of their visits.)

Ask the women for their perceptions about the impact that the programme might be having on the way in which the staff at the antenatal clinics are doing their job.

Discuss with the women whether or not the Hati Punguzo programme has affected their views about sleeping under an ITN while pregnant and/or their views about malaria in pregnancy. Also ask them to discuss their perceptions about why the government has undertaken the programme.

Ask the women to describe what they think are the good things about the programme and any problems or concerns that they have with it.

End the discussion with an invitation to the women to ask any additional questions or mention any concerns that we might try & address.
