# Supplementary material for: Vouchers for scaling up insecticide-treated nets in Tanzania: Methods for monitoring and evaluation of a national health system intervention
Source: BMC Public Health. 2008 Jun 10;8:205. doi: 10.1186/1471-2458-8-205 (PMC2442068; doi:10.1186/1471-2458-8-205)
Supplement: Additional file 5 — Health worker interview guide. [file 1471-2458-8-205-S5.pdf]

Ifakara Health Research and Development Centre and London School of Hygiene and Tropical Medicine

Monitoring and Evaluation of the Tanzania National Voucher Scheme for insecticide-treated nets

Health worker group interview topic guide

SECTION 1: IDENTIFIERS

	Variable Code		Variable Code
Date<br><br>|__|__| / |__|__| / |__|__|__|__|			
District<br><br>|__|__|__|__|__|__|__|__|__|__|__|			
Facility Code |__|__|__|		Group ID |__|__|__|	
Facility Type |__| (1)Dispensary (2)Health Centre (3)Hospital		Interviewer ID |__|__|	

TOPIC GUIDE

I'd like to start by asking just a few general questions about this health facility, the services you offer and the staff that are employed here.

How many days a week do you run an antenatal clinic here? (Probe for how many days a week/month and which days)
On a usual antenatal clinic day roughly how many women attend the clinic? Does this vary much?

How many nurses work in the antenatal clinic? (probe – is it always the same number, or some days is it more and others less – what is the maximum and minimum number?)
At this clinic is your antenatal clinic fully staffed – do you have as many staff as you are supposed to have or are some positions vacant?
In your opinion do you think you have enough nurses here to run the antenatal clinics? Why do you think this? (*try and get the participants to discuss the reason for their answers*)

In your opinion how far into the pregnancy do you think women should make their first visit to an antenatal clinic? Why do you think they should come at this time (probe for why not earlier or later)? What advice do you give women about when they should first come and how often they should come to an antenatal clinic during pregnancy?

What services, advice and treatments do you offer to women on their first ANC visit? *(Try and get the participants to agree on a list of what they offer and do BUT also make note of any disagreements)*

Pregnant Women Voucher (PWV)

How long has the PWV been operating in that clinic?
Have any of you received training in the PWV scheme?

For those that have received training probe for: who trained you, what did the training consist of (how long did it take) and how long ago was the training? Have there been any training updates or new information provided about how the scheme should work, and if so when and by whom?
Do new staff get trained in PWV when they arrive? If so then by whom?

How many of you are involved in distributing the vouchers?

For those that are involved in distributing the vouchers please can you tell me about how you decide when a women should be given a voucher (probe for: when they show they can buy a net, any woman on the first visit, etc)?
Are there ever times when you have not given a voucher to every woman on her first visit? (probe for: is it sometimes on the second or subsequent visit?)
What are the criteria for these decisions - what are the reasons that you don't necessarily give a voucher to every woman on their first visit?

Have you ever run out of the PWV at this clinic? If so then what did you do and how long was it before you got more vouchers (If happened more than once then ask how long it took to get new PWV each time)?

Do you know where the women go to redeem their PWV? Is there a shop nearby that sells nets? What do you tell women about where they can go to redeem their PWV and do you give them any information on how much they should expect to pay?

What is the 'value' of the PWV?

I'd like now to know a little more about your experiences with the scheme, please could you tell me what you think about the PWV scheme, if you think it has affected your work and if so then how has it affected you?

NB – try to make sure you get information on:
If they ever get pressure from others (people other than non-pregnant women) to give vouchers to them. If they do get pressure then who gives this pressure, who do they want vouchers for & how do they deal with this type of situation?
Do they think it has increased their workload and if so then why and in what way?

Can any of you tell me of any examples of difficult decisions you have had to make with PWV?

Outreach Services
Do you provide Outreach/Mobile RCH services from this clinic? (Probe for pregnant women and under fives- both, none or one or the other)

If yes: In how many locations are these services provided? How often are the services provided at each location?
What services do you offer at these clinics? Probe for: do you take PWV?

If vouchers are taken on outreach then ask: what do the women who receive vouchers at the outreach/mobile RCH services do about redeeming the voucher? How close is the nearest shop selling nets? How easy is it to reach that outlet? Is it in a place they would visit for other reasons (market, to receive other RCH tests etc)?
If only one or neither voucher is taken on outreach then probe: why don't you take the voucher(s)? Are there any problems with taking vouchers to the outreach/mobile services? What are these problems & how do you think they could be addressed (solved)? If only one type of voucher taken then probe: why don't you take both types of voucher?

Do you think there are many women in the catchment area of this clinic (including the outreach areas) who never use RCH services when they are pregnant or after their children are born? If there are then in your opinion what are their reasons for not attending?

General Effects of PWV

Overall do you think the programme has had any effect on the timing of a woman's first visit to the ANC? If yes: what effect?

Has the introduction of the PWV had any other effects on (amount of work, relationship with the community etc)

Do you think this scheme can carry on like it is? Do you think it should be sustained and what are the reasons for your answers?

Overall what are your views about voucher programme (good and bad things)?

Do you have any questions you would like to ask us?

Thank you for taking time to help in this survey, you thoughts, experiences and opinions are very valuable to us
