# Supplementary material for: Vouchers for scaling up insecticide-treated nets in Tanzania: Methods for monitoring and evaluation of a national health system intervention
Source: BMC Public Health. 2008 Jun 10;8:205. doi: 10.1186/1471-2458-8-205 (PMC2442068; doi:10.1186/1471-2458-8-205)
Supplement: Additional file 6 — Retail survey questionnaire. [file 1471-2458-8-205-S6.pdf]

Tanzania National Voucher Scheme for insecticide treated nets
RETAIL CENSUS QUESTIONNAIRE 2007/8

Ifakara Health Research and Development Centre *in collaboration with*
Ministry of Health, Tanzania and London School of Hygiene and Tropical Medicine
Version 1: 27 Sept 2007

OUTLET CODE [ ]

**C1**	Interviewer initials	**[ ]**
**C2**	Date of Interview (dd/mm/yyyy)	|____|/|____|/|__|__|__|__|
**C3**	District name (write)	
**C4**	District code	|__|__|
**C5**	Ward Name	
**C6**	Ward code	☐☐☐☐
**C7**	Name of outlet	

**C8**	Name of respondent	
**C9**	Name of shop owner (if different from above)	
**C10**	Are you the:<br>1 = Shop/kiosk owner<br>2 = Shop/kiosk assistant<br>3 = self- employed<br>4 = Family member<br>5 = Other (*Specify*)	**[ ]**
**C11**	Do you sell?<br>1= Retail only<br>2 = Wholesale only<br>3 = Wholesale and retail<br>9= Don't know	**[ ]**

C12
What goods are on sale in this outlet today?

**INTERVIEWER:** ***Against each item write 1 = Yes, 2 = No or 9 = Don't know***	
**ITN Products and Drugs**	
Nets (without insecticide)	**[ ]**
Net kits (net and Insecticide)	**[ ]**
Insecticide - Ngao	**[ ]**
Other insecticide apart from Ngao, *specify*	**[ ]**
Anti-Malarial Drugs	**[ ]**

Tanzania National Voucher Scheme for insecticide treated nets
RETAIL CENSUS QUESTIONNAIRE 2007/8

Ifakara Health Research and Development Centre *in collaboration with*
Ministry of Health, Tanzania and London School of Hygiene and Tropical Medicine
Version 1: 27 Sept 2007

Drugs (other than anti-malarial drugs)	[ ]
Mosquito coil	[ ]
Insecticide sprays (eg Doom)	[ ]
Insect repellents	[ ]
**Food, Drink and Groceries**	
Fresh fruit and vegetables	[ ]
Fresh meat	[ ]
Loose food by weight (e.g. maize flour, sugar, beans, dried fish)	[ ]
Pre-packaged foods (e.g. packet tea, canned foods, jars of jam)	[ ]
Bottled drinks (e.g. sodas and beer)	[ ]
Cleaning products (e.g. soap powder, washing soap)	[ ]
Cosmetics	[ ]
**Clothing and Textiles**	
Ready made clothes	[ ]
Rolls of cloth	[ ]
Kanga	[ ]
Kitenge	[ ]
Bags or suit cases	[ ]
Bedcovers, blankets or sheets	[ ]
Mattresses	[ ]
Sandals (flip flops)	[ ]

**Others**	
General household goods (e.g. plasticware, bowls, pans, pots)	[ ]
Hardware (e.g. tools, cement, iron sheeting)	[ ]
Stationary (e.g. school books, pens, paper)	[ ]
Watches, clocks, jewellery	[ ]

Radio	**[ ]**
Umbrella	**[ ]**
Vehicle/bicycle spare parts	**[ ]**

**ITN and related product sales**		
**C13**	Do you normally sell unbundled mosquito nets?<br>1= Yes<br>2 = No<br>9 = Don't know	**[ ]**
**C14**	Do you normally sell mosquito nets packaged with insecticide?<br>1 = Yes<br>2 = No<br>9 = Don't know	**[ ]**
**C15**	Do you normally sell long-lasting treated nets or Magic Power nets? 1= Yes<br>2 = No<br>99 = Don't know	**[ ]**
**C16**	Do you normally sell insecticide for treating mosquito nets<br>1 = Yes<br>2 = No<br>9 = Don't know	**[ ]**
**INTERVIEWER: If the respondent does not sell any nets or insecticide products, go to C28**		

**Questions C17- C27 are for retailers selling ITN products**		
**C17**	How long have you been in the business of selling unbundled nets?<br>1 = Less than 6 months,<br>2 = 6 to 12 months,<br>3 = 1 to 3 years<br>4 = 4 to 6 years<br>5 = More than 7 years<br>8 = Not applicable/ Don't sell these items<br>9 = Don't know	**[ ]**
**C18**	How long have you been in the business of selling net kits?<br>1 = Less than 6 months,<br>2 = 6 to 12 months,<br>3 = 1 to 3 years<br>4 = 4 to 6 years<br>5 = More than 7 years<br>8 = Not applicable/Don't sell these items<br>9 = Don't know	**[ ]**
**C19**	How long have you been in the business of selling long lasting treated nets or Magic Power nets<br>1 = Less than 6 months<br>2 = 6 to 12 months<br>3 = 1 to 3 years<br>4 = 4 to 6 years<br>5 = More than 7 years<br>8 = Not applicable/Don't sell these items<br>9 = Don't know	**[ ]**
**C20**	How long have you been in the business of selling insecticide?<br>1 = Less than 6 months<br>2 = 6 to 12 months	**[ ]**

	3 = 1 to 3 years<br>4 = 4 to 6 years<br>5 = More than 7 years<br>8 = Not applicable/Don't sell these items<br>9 = Don't know	
**C21**	When do you stock/sell nets?<br>1 = All year round<br>2 = Part of the year<br>3 = On request<br>4 = Other (specify)<br>8 = Not applicable/Don't sell these items<br>9 = Don't know	**[ ]**
**C22**	Please give the NAME and LOCATION of the supplier of mosquito nets (write here)<br><br>*Write '99' if not known or '88' if not applicable/don't sell these items*	
**C23**	Does your net supplier deliver to your outlet?<br>1 = Yes<br>2 = No<br>*8 = Not applicable/Don't sell these items*<br>9 = Don't know	**[ ]**
**C24**	Give the NAME and LOCATION of your supplier of insecticide<br><br>*Write 99 if not known or '88' if not applicable/don't sell these items*	
**C25**	Does your insecticide supplier deliver to your outlet?<br>1 = Yes<br>2 = No<br>*8 = Not applicable/Don't sell these items*<br>9 = Don't know	**[ ]**

**C26**	What brand of nets do you have in stock today?<br>*Against each item write 1 = Yes, 2 = No or 9 = Don't know*	
	nets	**[ ]**
	net	**[ ]**
	net	**[ ]**
	Net	**[ ]**
	ABC	**[ ]**
	B52	**[ ]**
	Motex	**[ ]**
	B58	**[ ]**
	Olyset	**[ ]**
	Other (please specify)	**[ ]**

INTERVIEWER: If answer is 'No' for all brands of net, Go to C31

C27 Please tell us more about the prices of <u>bundled net kits</u> (nets plus insecticide) you have in stock today?

INTERVIEWER: Here we want you to record the lowest and highest price for each size of net listed below.
"Cost price" refers to the price paid by the shop owner to the wholesaler for each net.
*"Retail price" refers to the price paid by a customer for a net **<u>without</u>** a voucher*
*"Voucher price" refers to the **top-up amount** which is paid by customers who buy a net with a voucher*

Notes:
- *It does not matter whether the net is round or square*
- *Where there is only **one** price for a net size please write the **same** price in the Low and High boxes.*
- *Where a net size is **<u>not stocked</u>** please write '8888' in the Low and High boxes.*
- *If any price is **<u>not known</u>** write '9999'.*
- ***Do not** leave any boxes blank.*

Size (round or square)	Cost price		Retail Price		Voucher price			
	Low	High	Low	High	Children's vouchers		Pregnant women voucher	
					Low	High	Low	High
3.5X6X7								
4x6x5								
4X6X7								
5X6X7								
6X6X5								
6X6X7								
8X6X7								

INTERVIEWER: Now go to C31

Reasons for not selling ITN products (for outlets not currently selling nets)		
**C28**	Have you ever sold mosquito nets (bundled or unbundled) in the past?<br>1 = Yes<br>2 = No<br>9 = Don't know	**[ ]**<br>**If 'No' or 'Don't know' go to C30**
**C29**	Please give reason(s) why you stopped selling	**[ ]**<br>**Go to C31**
**C30**	Please give reason(s) why you have never sold these products.	**Go to C31**

**Knowledge and involvement in the TNVS**		
**C31**	Have you heard of the voucher scheme for ITNs?<br>1 = Yes<br>2 = No<br>9 = Don't know	**[ ]**<br>**If 'No' or 'Don't know' skip to C43**
**C32**	Do you currently accept vouchers for ITNs<br>1 = Yes<br>2 = No<br>9 = Don't know	**[ ]**<br>**If 'No' or 'Don't know' skip to module C40**
**C33**	When did you start accepting vouchers?<br>(mm/yyyy)<br><br>*Write 9 if not known*	|__|__|/|__|__|__|__|
**C34**	How often do you redeem your vouchers?<br>1 = Weekly<br>2 = Every 2 weeks<br>3 = Monthly<br>4 = Other (please specify)<br>5 = Have not yet redeemed<br>9 = Don't know	**[ ]**
**C35**	How often would you like to redeem your vouchers?<br>1= Weekly<br>2 = Every 2 weeks<br>3 = Monthly<br>4 = Other (please specify)<br>9 = Don't know	**[ ]**

Tell the respondent that you now want them to think about the **last time** they redeemed vouchers. If the respondent has not yet redeemed any vouchers skip to C43		
**C36**	When was the last time you redeemed some vouchers?<br>1 = Less than 1 week ago<br>2 = 1-2 weeks ago<br>3 = 2-4 weeks ago<br>4 = More than one month ago<br>99 = Don't know	**[ ]**
**C37**	Approximately how many vouchers did you redeem on this occasion? (write number)<br><br>*Write 9999 if not known*	**[ ]**
**C38**	Is the redemption process satisfactory or not satisfactory?<br>1= Satisfactory<br>2 = Not satisfactory<br>9 = Don't know/can't say	**[ ]**<br>**If 'satisfactory' or 'don't know' skip to C43**

**C39**	If the redemption process is not satisfactory please explain why	**Now go to C43**

**For shops not currently accepting vouchers**		
**C40**	Have you ever accepted vouchers in the past and then stopped?<br>1= Yes<br>2 = No<br>9 = Don't know	**[ ]**<br>**If 'No' go to C42**
**C41**	Why do you not currently accept vouchers?<br><br>*(write reason here)*	
**C42**	Would you like to be involved in a voucher scheme in future?<br>1= Yes<br>2 = No<br>99= Don't know	**[ ]**

**Other outlets selling ITN products in the area**		
**C43**	Do you know of any outlets in your area that sell nets and/or insecticides?<br>1= Yes<br>2 = No<br>9 = Don't know	**[ ]**<br>**If 'No' or 'don't know' skip to C45**
**C44**	Give their names and physical addresses	

Tanzania National Voucher Scheme for insecticide treated nets

RETAIL CENSUS QUESTIONNAIRE 2007/8

Ifakara Health Research and Development Centre *in collaboration with*
Ministry of Health, Tanzania and London School of Hygiene and Tropical Medicine

Version 1: 27 Sept 2007

Inform the respondent that you are now going to write down details of the shop and check what point of sales materials are on display		
**C45**	Are point-of-sales materials on display for Nets?<br>1= Yes<br>2 = No	**[ ]**
**C46**	Are point-of-sales materials on display for Ngao?<br>1= Yes<br>2=No	**[ ]**
**C47**	Are point-of-sales materials on display for TNVS?<br>1= Yes<br>2=No	**[ ]**
**C48**	Location of outlet *(Give as much detail as possible)*	shoploc
**C49**	Write down the GPS co-ordinates of outlet	*E*_______ ^{0} ______.__________<br><br>*S* ________ ^{0} _____.__________
**C50**	Type of outlet<br>1 = Machinga<br>2 = Kiosk/Kibanda<br>3 = Duka<br>4 = Supermarket<br>5 = Pharmacy (Part 1)<br>6 = Duka La Dawa (Baridi – Part 2)<br>7 = Other (please specify)<br>9 = Don’t know/can’t tell	**[ ]**
**C51**	Status of Interview<br>1 = Interview was completed<br>2 = Outlet is closed temporarily<br>*(take note to come back later)*<br>3 = Outlet is closed permanently<br>*(report to supervisor)*<br>4 = Respondent is busy<br>*(make appointment to come at convenient time)*<br>5 = No cooperation<br>6 = Other (*specify*)	**[ ]**

Thank the Respondent
