# Supplementary material for: Vouchers for scaling up insecticide-treated nets in Tanzania: Methods for monitoring and evaluation of a national health system intervention
Source: BMC Public Health. 2008 Jun 10;8:205. doi: 10.1186/1471-2458-8-205 (PMC2442068; doi:10.1186/1471-2458-8-205)
Supplement: Additional file 7 — Voucher tracking survey, Form 1 (recipient found). [file 1471-2458-8-205-S7.pdf]

Tanzania National Voucher Scheme for insecticide-treated nets
VOUCHER TRACKING SURVEY 2007

Ifakara Health Research and Development Centre in collaboration with Ministry of Health, Tanzania and London School of Hygiene and Tropical Medicine

FORM 1: TO BE USED AFTER IDENTIFYING THE VOUCHER RECIPIENT

(USE FORM 2 FOR A MISSED VOUCHER RECIPIENT)

A. Information about the voucher

A1	District (write full name of the district) |__|__|__|__|__|__|__|__|__|__| |__|__|__||__||__|	
A.2	Type of health facility<br>1= Dispensary<br>2= Health centre<br>3= Hospital	|__|
A.3	MEDA records indicated that:<br>3= Voucher has been redeemed<br>2= Stub has been returned but voucher not redeemed<br>3= Stub has been returned and voucher redeemed	
A.4	What was available?<br>1= Voucher only<br>2= Stub only<br>3= Voucher and stub<br>4= Voucher number only	|__|
A.5	Name of the health facility	____________________
A.6	Interviewer initials	|__|__|
A.7	Voucher number	|__|__|__||__||__||__||__||__|
A.8	Date written on the voucher	|__|__|/|__|__|/|__|__|__|__|
A.9	Name	____________________
A 10	Ward	____________________
A.11	Village	____________________
A.12	Ten cell leader/Street chairperson	____________________

B. Information about the voucher recipient *(read the consent form to her)*

B.1	Consent given?<br>(1 = Yes, 2 = No)	|__|
B.2	Date of interview	|__|__|/|__|__|/|__|__|__|__|
B.3	What is your name?	
B.4	What is your current age?	|__|__|
B.5	Sex (1 = Male, 2 = Female)	|__|
B.6	Village	____________________
B.7	Sub village	____________________
B.8	Ten cell leader	____________________
B.9	Location of the house?<br>1 = Urban<br>2 = Village<br>3 = Farm	|__|

B.10	Are you the head of the household?<br>(1 = Yes 2 = No)	|__|
B.11	How many completed years of education?<br>*(write the number of completed years of education, if less than a year write 00)*	|__|__|
B.12	What is your economic activity?<br>1 = Farming/fishing/forest related activities<br>2 = Non -farming casual work<br>3 = Prefessional/clerical<br>4 = Service provision (tailoring, carpentry etc)<br>5 =Petty trade (vegitables or other things)<br>6= Bussiness<br>7= House maid/maker<br>8= Student<br>9= None	|__|
B.13	Marital status<br>1. Single<br>2. Married<br>3. Separated/divorced<br>4. Widow<br>5. Cohabiting	|__|
B.14	How many children do you have?	|__||__| If 0 GO TO C.1
B.15	How many children are less than five years old?	|__|
B.16	What is the birth date of your youngest child?	|__|__|/|__|__|/|__|__|__|__|
B.17	*Interviewer: Is the woman still pregnant?*<br>(1= Yes, 2=No)	|__|

C Knowledge and use of voucher and net

C.1	Have you ever heard about the voucher programme for buying a net at a subsidised price?<br>(1 = Yes, 2 = No)	|__| IF NO GO TO C.3
C.2	Where did you hear about it for the first time?<br>01 = RCH clinic/health facility<br>02 = Shop<br>03 = Family member<br>04 = Neghbour/friend<br>05 = Radio<br>06 = Peformance by theatre group or road show<br>07 = Village government<br>08 = Do not remember<br>09 = I do not know<br>10 =Other, (mention)	|__||__| ____________________
C.3	Have you ever used ANC services within the past 2 years?<br>(1 = Yes, 2 = No)	|__|
C.4	*(Show her the voucher and ask)*<br>Did you receive this kind of a voucher from the RCH clinic?<br>(1 = Yes, 2 = No)	|__| IF NO GO TO C.8
C.5	*(Request the respondent to give you her ANC card)*<br>Is the voucher number written on the ANC card?<br>(1 = Yes, 2 = No)	|__|
C.6	How many vouchers have you received?	|__|__|

	(Interviewer: If the woman has received more than one voucher, focus on the specific voucher. Use the date and place of the issue as indicated in the first section to help the respondent to understand the index voucher).	
C.7	What made you receive the voucher?<br>1 = Being pregnant<br>2 = I had a child less than one year<br>3 = Other (explain)	|__| ______________________<br>NOW GO TO C.9
C.8	What do you think are the reasons that made you not be given the voucher?<br>______________________<br>NOW GO TO C.30	
C.9	Did you pay for the voucher?<br>(1 = Yes, 2 = No)	|__| IF NO GO TO C.11
C.10	How much did you pay? (Tsh)	|__|__|__|__|
C.11	Did you use the voucher to buy a net?<br>(1 = Yes, 2 = No)	|__| IF YES GO TO C.16
C.12	Why didn't you use your voucher to buy a net?<br>1 = Gave it to another person<br>2 = I already have a net<br>3 = I did not have money to parchase the net<br>4 = I lost it<br>5 = I sold it to someone<br>6 = Nets are sold too far from here<br>7 = I do not know where to buy the net<br>8 = Other (explain)	|__| IF 1 OR 4 GO TO C30<br>IF 2 OR 3 OR 6 OR 7 OR 8 GO TO C13<br>IF 5 GO TO C15<br>______________________
C.13	Do you still have the voucher? (1 = Yes, 2 = No)	|__| IF YES GO TO C14<br>IF NO GO TO C.30
C.14	*(Interviewer: Request her to show you the voucher)*<br>Did you see the voucher?<br>(1 = Yes, 2 = No)	|__| NOW GO TO C30
C.15	For how much did you sell the voucher?	|__|__|__|__| NOW GO TO C30
C.16	Did you buy the net while pregnant or after delivery?<br>1 = While pregnant<br>2 = After delivery	|__|
C.17	When did you buy the net? (*Month and year*)	|__||__|/|__||__|
C.18	Where did you buy this from?<br>1 = Shop<br>2 = RCH clinic<br>3 = Other, explain	|__| ______________________
C.19	How long did you take to travel to the place where you bought the net? *(indicate minutes)*	|__|__|__|

C.20	How much did you pay for transport? (Tsh) *(interviewer write 0 if did not pay anything)*	|__|__|__|__|
C.21	How much did you pay for the net? *(on top of the voucher)*	|__|__|__|__|
C.22	What is the size of the net? *("feet"write y x z)*	
C.23	Do you still have the net?<br>(1 = Yes, 2 = No)	|__| IF YES GO TO C24<br>IF NO GO TO C25
C.24	*(interviewer: ask if you could see the net)*<br>Did you see the net?<br>(1 = Yes, 2 = No)	|__| NOW GO TO C27
C.25	Where is that net now?<br>1 = Stolen<br>2 = Burnt<br>3 = Lost<br>4 = Sold<br>5 = Gave it to someone else<br>6 = Gave it to the one who paid<br>7 = Other, explain	|__| IF 4 GO TO C26<br>OTHERWISE GO TO C30<br>________________
C.26	At what price did you sell the net?	|__||__||__||__|
C.27	Was the net used last night?<br>(1= Yes, 2= No)	|__|
C.28	Who slept under the net last night?<br>(1= Mentioned 2= Not mentioned)	
	Myself	|__|
	Infant	|__|
	Child under five years (but over one year)	|__|
	My husband	|__|
	Other family member	|__|
	Other, mention	☐<br>________________
C.29	Who slept under the net when you were pregnant? *(If still pregnant or purchased the net alter delivery do not ask this questioni)*<br>(1= Mentioned 2= Not mentioned)	
	Myself	|__|
	Infant	|__|
	Child under five years (but over one year)	|__|
	My husband	|__|
	Other family member	|__|
	Other, mention	☐<br>________________
C.30	Did you receive any net from the clinic within the past two years?<br>(1 = Yes, 2 = No)	|__| If NO GO TO C.33

C.31	What is the name of the clinic?	
C.32	How much money did you pay? (Tsh) *(write 0 if the net was given for free)*	|__||__||__||__|
C.33	What are your views about the good aspects or weakness of the voucher programme. ________________ ________________	

D Insecticide Re-treatment Kit (IRK) – For children under one year

D1	Could you mention the names of the children under one year who are under your care?	Name of the child (1) ________________	Name of the child (2) ________________
D2	Have you taken (name) to the RCH clinic for vaccination?<br>(1= Yes, 2=No)	|__|	|__|
D3	May I see the child health card?<br>*Interviewer record:*<br>*1= Card seen*<br>*2= Card not seen*	|__|	|__|
D4	Did you receive a Hati Punguzo insecticide re-treatment kit?<br>(1=Yes, 2=No)	|__|	|__|
D5	Interviewer: Is the IRK recorded on the card?<br>(1=Yes, 2=No)	|__|	|__|
D6	How many times did you receive a kit?	|__|	|__|
D7	Have you used a Hati Punguzo IRK to treat a net?<br>(Yes=1, 2=No)	|__|	|__|

E Household characteristics

E.1	Name of the head of hosuehold?	________________
E.2	Sex of the head of household?<br>(1 = Male, 2 = Female)	|__|
E.3	Age of the head of household (years)	|__|__|
E.4	What is the number of completed years of education of the head of household? (write the completed number of years, if less than one year write 00 and if do not know write 99)	|__|__|
E.5	What is your economic activity?<br>1 = Farming/fishing/forest related activities<br>2 = Non -farming casual work<br>3 = Prefessional/clerical<br>4 = Service provision (tailoring, carperntry etc)<br>5 =Petty trade (vegitables or other things)<br>6= Bussiness<br>7= Household maid/maker<br>8= Student<br>9= None	|__|

E.6	How many people live in this household?	|__|__|
E.7	How many children are under 15 in this household?	|__|__|
E.8	How many children are under 5 in this household?	|__|
E.9	How many nets does this household have?	|__|
E.10	How many people in this household slept under a net last night?	|__|__|
E.11	How many children under five slept under a net last night?<br>*(Do not ask if there are no children under five)*	|__|
E12	Is this house rented?<br>1= Yes<br>2= No<br>3= Other, explain	|___|
E13	What type of toilet does this household use?<br>1 = Flush toilet<br>2 = Latrine<br>3 = No toilet	|__|
	Is there any person in this household who owns the following?	
E14	Radio<br>(1 = Yes, 2 = No)	|__|
E15	Bicycle<br>(1 = Yes, 2 = No)	|__|
E16	Mobile phone<br>(1 = Yes, 2 = No)	|__|
E17	How many chicken or ducks does this household own?	|__|__|__|__|
E18	How many livestock such as cows, sheep or goat does this household own?	|__|__|__|__|
E19	Is this household connected to the electricity?<br>(1 = Yes, 2 = No)	|__|
E 20	What is the roofing material?<br>1 = Iron/tiles<br>2 = Thatch<br>3 = Other, explain	|__| ____________________

Thank you
