# Supplementary material for: Vouchers for scaling up insecticide-treated nets in Tanzania: Methods for monitoring and evaluation of a national health system intervention
Source: BMC Public Health. 2008 Jun 10;8:205. doi: 10.1186/1471-2458-8-205 (PMC2442068; doi:10.1186/1471-2458-8-205)
Supplement: Additional file 8 — Voucher tracking survey, Form 2 (recipient not found). [file 1471-2458-8-205-S8.pdf]

Tanzania National Voucher Scheme for insecticide-treated nets
VOUCHER TRACKING SURVEY 2007

Ifakara Health Research and Development Centre in collaboration with Ministry of Health, Tanzania and London School of Hygiene and Tropical Medicine

QUESTIONNAIRE FOR RECIPIENTS WHO ARE NOT FOUND
(Use Form 1 for the found recipients)

A. INFORMATION ABOUT THE VOUCHER

A1	District (write full name of the district)	|__|__|__|__|__|__|__|__|__|__| |__|__|__||__||__|
A.2	Type of health facility<br>1= Dispensary<br>2= Health centre<br>3= Hospital	|__|
A.3	MEDA records indicated that:<br>3= Voucher has been redeemed<br>2= Stub has been returned but voucher not redeemed<br>3= Stub has been returned and voucher redeemed	
A.4	What was available?<br>1= Voucher only<br>2= stub only<br>3= Voucher and stub<br>4= Voucher number only	|__|
A.5	Name of the health facility	________________________
A.6	Interviewer initials	|__|__|
A.7	Voucher number	|__|__|__||__||__||__||__||__|
A.8	Date written on the voucher	|__|__|/|__|__|/|__|__|__|__|
A.9	Name	________________________
A 10	Ward	________________________
A.11	Village	________________________
A.12	Ten cell leader/street chairperson	________________________

B. REASONS FOR MISSING

B.1	Did you get the person you were tracking?<br>(1 = Yes 2 = No)	|__| IF YES, FILL PWV
B.2	Reasons for failure to interview the voucher respondent<br>1 = Travelled out of the district<br>2 = Travelled and nobody knows where she is<br>3 = Nobody knows that person but the ten cell leader is known<br>4 = Both the person and the ten cell leader are not known<br>5 = Died<br>6 = Migrated out<br>7 = Other, mention	|__| **END**<br>________________________
